# “One Health” perspective on prevalence of co-existing extended-spectrum *β*-lactamase (ESBL)-producing *Escherichia coli* and *Klebsiella pneumoniae*: a comprehensive systematic review and meta-analysis

**DOI:** 10.1186/s12941-023-00638-3

**Published:** 2023-09-22

**Authors:** Tsepo Ramatla, Tshepo Mafokwane, Kgaugelo Lekota, Maropeng Monyama, George Khasapane, Naledi Serage, Jane Nkhebenyane, Carlos Bezuidenhout, Oriel Thekisoe

**Affiliations:** 1 Unit for Environmental Sciences and Management, North-West University, Potchefstroom, 2520 South Africa; 2 Department of Life and Consumer Sciences, University of South Africa, Florida, 1710 South Africa; 3 Department of Life Sciences, Central University of Technology, Bloemfontein, 9300 South Africa

**Keywords:** One health, ESBL, *E. coli*, *K. pneumoniae*, Global, meta-analysis

## Abstract

**Background:**

The *Escherichia coli (E. coli)* and *Klebsiella pneumoniae (K. pneumoniae)* bacterial isolates that produce extended-spectrum *β*-lactamases (ESBLs) contribute to global life-threatening infections. This study conducted a systematic review and meta-analysis on the global prevalence of ESBLs in co-existing *E. coli* and *K. pneumoniae* isolated from humans, animals and the environment.

**Methods:**

The systematic review protocol was registered in the International Prospective Register of Systematic Reviews (PROSPERO) [ID no: CRD42023394360]. This study was carried out following the preferred reporting items for systematic reviews and meta-analyses (PRISMA) guidelines. One hundred and twenty-six eligible studies published on co-existing antibiotic resistance in *E. coli* and *K. pneumoniae* between 1990 and 2022 were included.

**Results:**

The pooled prevalence of ESBL-producing *E. coli* and *K. pneumoniae* was 33.0% and 32.7% for humans, 33.5% and 19.4% for animals, 56.9% and 24.2% for environment, 26.8% and 6.7% for animals/environment, respectively. Furthermore, the three types of resistance genes that encode ESBLs, namely *bla*_*SHV*_*bla*_*CTX−M*_,*bla*_*OXA*_, and *bla*_*TEM*_, were all detected in humans, animals and the environment.

**Conclusions:**

The concept of “One-Health” surveillance is critical to tracking the source of antimicrobial resistance and preventing its spread. The emerging state and national surveillance systems should include bacteria containing ESBLs. A well-planned, -implemented, and -researched alternative treatment for antimicrobial drug resistance needs to be formulated.

**Supplementary Information:**

The online version contains supplementary material available at 10.1186/s12941-023-00638-3.

## Background

The extended-spectrum *β*-lactamase (ESBL) produced by Enterobacteriaceae is a common source of antimicrobial resistance (AMR) in both animals and humans [1]. These ESBL-producing bacteria are often associated with multidrug resistance (MDR) against other antibiotic classes [2]. They develop resistance to third- and fourth-generation cephalosporins, as well as aztreonam, which is among the last medications available to treat infections caused by *Escherichia coli* and *Klebsiella pneumoniae* [1, 3–5]. However, they are not resistant to carbapenems or cephamycin [4].

*Escherichia coli* and *K. pneumoniae* are two examples of ESBL-producing Enterobacteriaceae (ESBL-E) that were the first to be linked to infections in the medical context [6, 7]. *Escherichia coli* and *K. pneumoniae* can cause a wide range of serious illnesses, which are becoming increasingly difficult to treat due to the development of antibiotic resistance [8]. Carbapenem is an antibiotic used as a last option to treat beta-lactamase Enterobacteriaceae transfer [9]. The spread of *E. coli* and *K. pneumoniae* extended spectrum cephalosporinase in animal populations, the environment, and the community proves that such bacterial strain are transmitted and persist outside of clinical settings [8, 10].

In the Enterobacteriaceae family of commensal or pathogenic bacteria, ESBLs are crucial antibiotic resistance determinants that are passed on through horizontal gene transfer [9]. Among several ESBL gene variants, the most prevalent and clinically relevant are *bla*_*TEM*_, *bla*_*SHV*_ (sulphydryl variable) and *bla*_*CTX−M*_, (cefotaxime-hydrolyzing β-lactamase) [4]. There are several families of ESBLs, including the *bla*_*TEM*s_-type ESBLs and the *bla*_*SHVs*_-type ESBLs, with variants that differ only by a few amino acid substitutions [4]. There are some unique characteristics in each of the ESBL families. The *bla*_*TEM−1*_, *bla*_*TEM−2*_ and their ESBL derivatives are usually carried by transposons like *Tn1*, *Tn2*, or *Tn3* [4, 11]. An association has been found between the spread of *bla*_*CTX−M*_-producing enzymes and *E. coli* belonging to the ST131 clonal group [12]. It has been reported that ST131 isolates contain several *bla*_*CTX−M*_ types, including *bla*_*CTX−M−15*_ [13]. The empirical treatment of ESBL-producing Enterobacteriaceae infections has become increasingly difficult [14].

The presence of ESBL-producing *E. coli* and *K. pneumoniae* in humans, animals, and the environment poses a public health threat. In addition, it is crucial to maintain current data on ESBL-producing *E. coli* and *K. pneumoniae* in health systems to minimize the consequences of ESBL-producing bacteria. A few systematic reviews have been published on ESBL-producing *E. coli* in animals, humans, and the environment in Bangladesh and South America [15, 16], as well as ESBL-producing *E. coli* and *K. pneumoniae* in the United States of America [17]. However, there is limited information on comprehensive data available to estimate the global prevalence of co-existing ESBL-producing *E. coli* and *K. pneumoniae* in animals, humans, and the environment. The current study is a systematic review and meta-analysis aimed at providing a comprehensive prevalence of co-existing ESBL-producing *E. coli* and *K. pneumoniae*, in animals, humans, and the environment based on available data published globally.

## Materials and methods

### Protocol registration

This study was registered in the International Prospective Register of Systematic Reviews (PROSPERO) with registration no: CRD42023394360.

### Study design and systematic review protocol

Using the Preferred Reporting Items for Systematic Reviews and Meta-analyses (PRISMA) guidelines [18], data extraction, screening, and analysis involved searching database systems for potentially pertinent articles, assessing their relevance, and evaluating whether they were acceptable for review.

### Search strategy

This study used several database systems, including ScienceDirect, PubMed, Google Scholar, and Scopus to search for articles published in English from 1 to 1990 until 28 November 2022. Literature searches were conducted using keywords comprising “ESBL, extended-spectrum beta-lactamase,” “extended-spectrum *β*-lactamase,” “Human,” “Animal,” “Environment,” “*Escherichia coli* or *Klebsiella pneumoniae*,” and “*E. coli* or *K. pneumoniae.*” The last search took place on 28 November 2022.

### Study selection

Journal article titles and abstracts were reviewed for suitability for the inclusion and exclusion criteria by three authors namely TR, TM, and OT. Two reviewers independently analysed all the studies from the search by title, abstract, and selected full texts, and a third reviewer arbitrated for any divergence. Full-text journal articles published in English were included, while reviews, conference abstracts, and book chapters were excluded. Journal articles were selected for full-text review if they investigated the co-existence of ESBL-producing *E. coli* and *K. pneumoniae* only and reported the number of isolates and the positive number of ESBL isolates from animals, humans, and the environment.

### Inclusion and exclusion criteria

Criteria for inclusion of studies were: (i) studies investigating the prevalence of ESBL-producing *E. coli* and *K. pneumoniae* in animals, humans, and the environment. The following exclusion criteria was used: (ii) no total number of isolates, (iii) no abstracts, reviews, experiments, and book chapters, and (iv) an unclear ESBL identification method.

### Data extraction

The following data were extracted and summarized by two authors independently (TR and TM) from the final selected studies: (1) author name, (2) year of publication, (3) location, (4) year of publication, (5) type of samples, (6) ESBL identification methods, (7) positive samples of *E. coli* and *K. pneumoniae* isolates from animals, humans and the environment, (8) total number of ESBL positive *E. coli* and *K. pneumoniae* isolates, and (9) genes encoded for antibiotic resistance (ESBL). The recorded data were compiled in Microsoft Excel for further analysis.

### Quality assessment of included studies

A critical appraisal tool developed by the Joanna Briggs Institute (JBI) was used to assess each study’s bias risk [19]. There are nine questions in this JBI instrument, which are: (1) Was the sample frame appropriate to address the target population? (2) Were study participants recruited in an appropriate way? (3) Was the sample size adequate? (4) Were the study subjects and setting described in detail? (5) Was data analysis conducted with sufficient coverage of the identified sample? (6) Were valid methods used for the identification of the condition? (7) Was the condition measured in a standard, reliable way for all participants? (8) Was there appropriate statistical analysis? (9) Was the response rate adequate, and, if not, was the low response rate managed appropriately? Each answer was scored 0 or 1 based on whether the answer was “yes” or “no”. NA was used to indicate that the question was not relevant to the study. Studies with scores of 7–8 indicated a low risk of bias, scores of 5–6 indicated a moderate risk of bias, and scores less than 5 indicated a high risk of bias. Only articles with low bias risks were included in this study.

### Data synthesis and data analysis

Comprehensive Meta-Analysis Software v.4.0 (https://www.meta-analysis.com/) was used to conduct the meta-analysis of the prevalence of ESBL-producing *E. coli* and *K. pneumoniae* in humans, animals, and the environment. The pooled prevalence of *E. coli* and *K. pneumoniae* isolates was measured and subgroup analysis was performed according to continent, country, year of publication, settings, ESBL-resistant genes, and samples collected. Random-effects models were used to generate forest plots showing the study-specific effect sizes with a 95% confidence interval (CI) for the pooled prevalence (PP). The I^2^ statistic was used to measure heterogeneity among studies. A value close to 0% indicates no heterogeneity, whereas a value close to 25%, 50%, and 75% corresponds to low, moderate, and high heterogeneity, respectively. The p-values correspond to the heterogeneities between studies from a Chi-squared test of the null hypothesis that there is no heterogeneity.

### Publication bias

Publication bias was measured using funnel plots to test for symmetry and this was further complemented using the Beg and Mazumdar rank correlation test and Egger’s regression test. For all the tests, a *p*-value of < 0.05 was considered statistically significant.

## Results

### Search and screening results

**Fig. 1:**
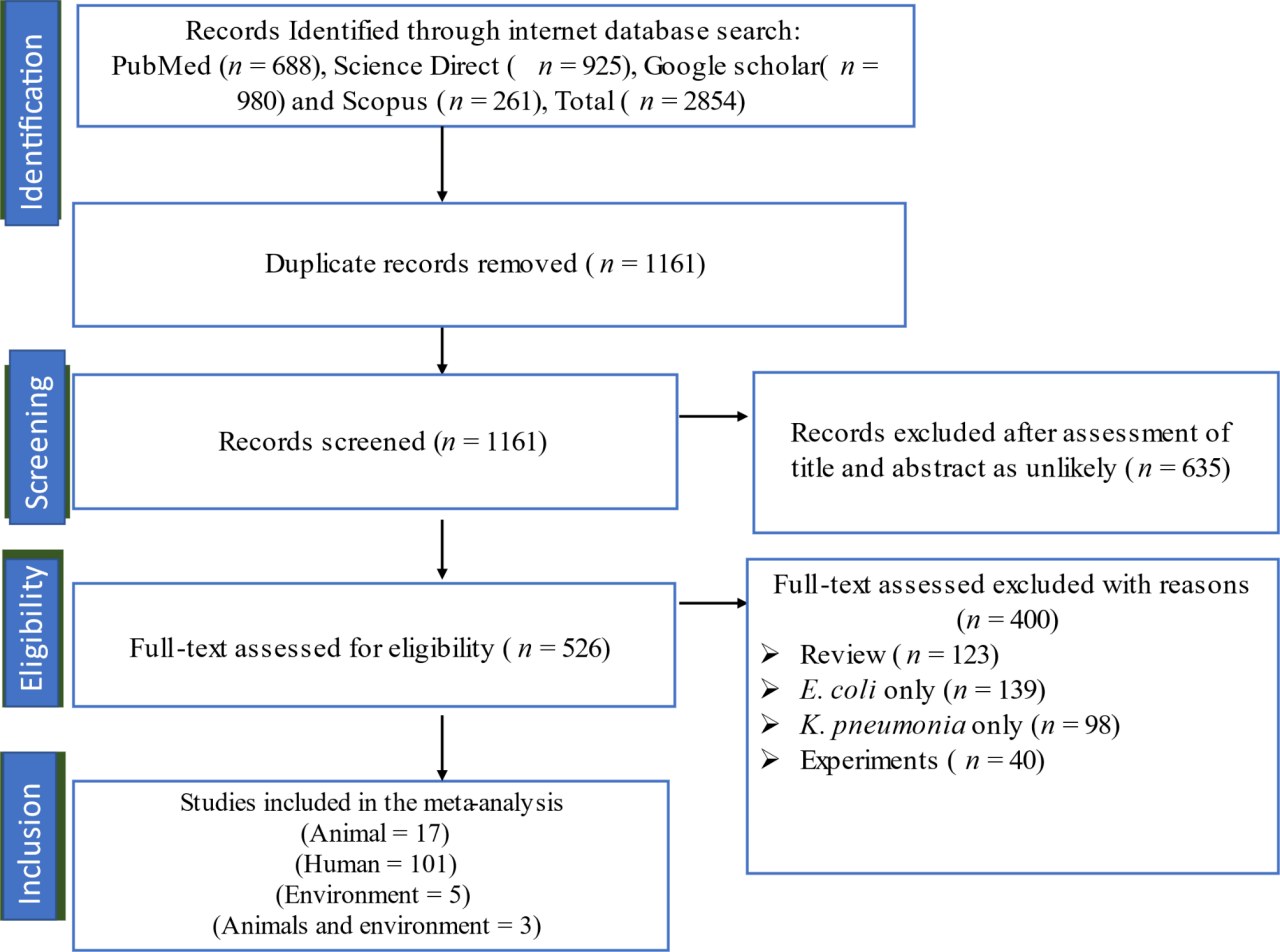
A PRISMA flow diagram illustrating the process of selecting studies

A total of 2854 studies were retrieved after the initial search was conducted across four databases, including 688 from PubMed, 925 from ScienceDirect, 980 from Google Scholar, and 261 from Scopus (Fig. 1). The abstracts of 1161 articles were screened based on the inclusion and exclusion criteria. A total of 526 studies were initially considered eligible and were thus subjected to full-text evaluation. After full-text examination, 126 animals (n = 17) were eligible for inclusion [humans (n = 101), environment (n = 5), animals and environment (n = 3)].

### Characteristics of eligible studies

All included studies were published from 1990 to 2022, with most of the studies having been conducted between 2000 and 2022. Out of four continents, Asia had the highest number of published studies (n = 80), followed by Africa (n = 37), Europe (n = 11), and the least was from North America (n = 8). The number of confirmed ESBL-producing *E. coli* and *K. pneumoniae* isolates in animals, human, and the environment ranged from 1 to 4706 globally. Numerous studies recorded the expression of antibiotic-resistant genes for the class of beta-lactams, including *bla*_*OXA*_ 20 (16%), *bla*_*CTX−M*_ 65(52%), *bla*_*TEM*_ 58 (46%), *bla*_*SHV*_ 65 (52%), *bla*_*CMY*_, 2 (2%), *bla*_*NDM*_ 2 (2%), *bla*_*PER*_ 2 (2%), and *bla*_*VEB*_, 2 (2%). However, six genes (*bla*_*DHA*_, *bla*_*FOX*_, *bla*_*MOX*_, *bla*_*KPC*_, *bla*_*VIM*_, and *blaampC*) were recorded once by different studies.

### Meta-analysis results on overall prevalence

Pooled prevalence estimates (PPE) of ESBL-producing *E. coli* and *K. pneumoniae* in animals, humans, and the environment, as well as a summary of the subgroup analysis, are shown in Tables 1 and 2, and 3. A total of 79,497 were confirmed as *E. coli*, while 37,998 were confirmed as *K. pneumoniae*. Only 18,923 and 8502 were confirmed as ESBL-producing *E. coli* and *K. pneumoniae*, respectively.

**Table 1:** Subgroup analysis of ESBL-producing *Escherichia coli* and *Klebsiella pneumoniae* in humans

Risk factors	Number of studies	Pooled estimates			Measure of heterogeneity			Publication bias
		Sample size	ESBL positive	I^2^ (95%CI)	Q value	I^2^	*Q*	Begg and Mazumdar rank *P*-value
**Overall Human**								
*E. coli*	101	75,064	17,513	33% (28.2–38.1)	11465.719	99.128	< 0.001	0.51290
* K. pneumoniae*	101	34,099	8165	32.7% (28.6–37.1)	4420.069	99.128	< 0.001	0.94853
**Human settings**								
**Hospital**								
*E. coli*	68	33,906	11,068	49% (41.1–57.4)	6538.749	98.975	< 0.001	0.70704
* K. pneumoniae*	68	17,106	4321	45.0% (38.8–53.4)	2695.361	97.514	< 0.001	0.38823
**Tertiary Hospital**								
*E. coli*	20	135,146	11,325	37.6% (20.9–58.0)	16771.387	99.887	< 0.001	0.06924
* K. pneumoniae*	20	28,333	4442	35.1% (22.8–49.7)	3742.403	99.519	< 0.001	0.15146
**Military Hospital**								
*E. coli*	3	4387	1373	42.3% (17.6–71.5)	368.625	99.457	< 0.001	0.11719
* K. pneumoniae*	3	2153	468	19.9% (12.8–29.7)	44.703	95.526	< 0.001	0.60151
**Study year**								
**1990–2000**						−		
*E. coli*	4	2719	192	10.9% (04.4–24.5)	116.215	97.419	< 0.001	0.49691
* K. pneumoniae*	4	1068	154	25.4% (7.1–60.0)	171.414	98.250	< 0.001	0.17423
**2000–2010**								
*E. coli*	27	11,107	2667	29.6% (21.5–39.1)	1764.542	98.527	< 0.001	0.40435
*K. pneumoniae*	27	7211	2405	39.7% (31.8–48.1)	989.685	97.372	< 0.001	0.19618
**2011–2020**								
*E. coli*	59	36,636	9422	35.8% (29.4–42.9)	6067.911	99.044	< 0.001	0.75857
* K. pneumoniae*	59	18,333	3545	28.9% (23.7–34.8)	2366.531	97.549	< 0.001	0.32342
**2021–2022**								
*E. coli*	11	12,719	5516	53.5% (41.1–65.5)	375.190	97.335	< 0.001	0.58579
*K. pneumoniae*	11	6524	1961	37.3% (28.7–46.8)	99.095	89.909	< 0.001	0.97893
**Samples**								
**Blood**								
*E. coli*	14	11,903	2875	22.7% (16.9–29.9)	641.879	97.975	< 0.001	0.62222
*K. pneumoniae*	14	7655	1278	34.2% (20.8–50.6)	1152.802	98.872	< 0.001	0.78430
**Faecal**								
*E. coli*	8	538	243	52.7% (29.3–74.9)	139.664	94.988	< 0.001	0.13765
* K. pneumoniae*	8	633	123	19.8% (9.9–35.8)	84.956	91.760	< 0.001	0.02595
**Urine**								
*E. coli*	24	16,547	2885	34.5% (24.6–45.9)	2047.148	98.876	< 0.001	0.92097
*K. pneumoniae*	24	3852		37.7% (28.7–47.7)	504.579	95.442	< 0.001	0.88169
**Stool and urine**								
*E. coli*	3	18,520	95	3.4% (1–10.4)	40.165	95.020	< 0.001	0.60151
*K. pneumoniae*	3	12,735	227	20.9% (4.6–59.1)	38.051	94.744	< 0.001	0.11719
**Various specimens**								
*E. coli*	33	23,970	7557	34.2% (26.1–43.3)	1996.713	98.397	< 0.001	0.43850
*K. pneumoniae*	33	13,261	3432	28.8% (22.1–36.6)	1087.196	97.057	< 0.001	0.75665
**Countries**								
**China**								
*E. coli*	3	859	363	39.7% (27.7–53.1)	23.425	91.462	< 0.001	0.60151
*K. pneumoniae*	3	877	165	23.4% (13.7–37.0)	16.911	88.173	< 0.001	0.60151
**India**								
*E. coli*	7	1506	455	35.2% (25.4–46.4)	85.911	93.016	< 0.001	0.22634
*K. pneumoniae*	7	970	303	36.3% (24.3–50.3)	89.011	93.259	< 0.001	0.92873
**Iran**								
*E. coli*	5	1433	404	42.9% (20.0–69.3)	259.145	98.456	< 0.001	0.32719
*K. pneumoniae*	5	436	221	55.0% (38.4–70.6)	44.200	90.950	< 0.001	0.43562
**Israel**								
*E. coli*	3	945	146	17.7% (9.7–30.1)	24.392	91.801	< 0.001	0.60151
*K. pneumoniae*	3	881	289	36.1% (28.7–44.3)	4.516	55.718	< 0.001	0.60151
**Korea**								
*E. coli*	9	2252	473	23.8% (10.8–44.4)	449.122	98.219	< 0.001	0.75445
*K. pneumoniae*	9			29.5% (19.5–41.9)	110.576	92.765	< 0.001	0.91697
**Nepal**					−	−	−	−
*E. coli*	4	1338	442	36.3% (13.3–68.0)	308.318	98.703	< 0.001	0.62421
*K. pneumoniae*	4	660	73	15.1% (8.3–25.7)	17.544	77.200	< 0.001	062421
**Nigeria**								
*E. coli*	12	1408	591	42.3% (25.1–61.6)	356.471	96.914	< 0.001	0.33705
*K. pneumoniae*	12	1107	313	27.0% (18.7–37.2)	106.094	89.632	< 0.001	0.68075
**Pakistan**								
*E. coli*	4	1815	587	34.3% (18.6–54.6)	174.838	98.284	< 0.001	1.0000
*K. pneumoniae*	4	1006	269	26.7% (12.2–48.8)	109.626	97.263	< 0.001	1.0000
**Saudi Arabia**								
*E. coli*	3	2271	441	34.6% (10.3–70.9)	297.555	99.328	< 0.001	0.60151
*K. pneumoniae*	3	1081	200	29.2% (9.8–61.1)	108.691	98.160	< 0.001	0.60151
**Taiwan**								
*E. coli*	6	1208	596	43.0% (31.1–55.7)	91.477	93.441	< 0.001	0.45269
*K. pneumoniae*	6	1182	568	44.4% (36.5–52.5)	38.247	84.313	< 0.001	0.29311
**Thailand**								
*E. coli*	6	16,792	6582	52.1% (42.0–62.0)	435.392	98.622	< 0.001	0.88062
*K. pneumoniae*	6	8336	8336	32.0% (22.3–43.6)	343.138	98.251	< 0.001	0.45269
**Turkey**								
*E. coli*	4	905	327	38.1% (25.7–52.3)	42.725	90.638	< 0.001	0.62421
*K. pneumoniae*	4	622	222	41.8% (31.8–52.4)	14.948	73.241	< 0.001	1.0000
**USA**								
*E. coli*	3	1173	203	23.2% (13.2–37.4)	10.706	81.319	< 0.001	0.117119
*K. pneumoniae*	3	286	139	55.7% (17.2–88.5)	76.316	97.379	< 0.001	0.60151
**Continent**								
**Africa**								
*E. coli*	28	3649	1291	44.3% (32.2–57.2)	859.940	96.860	< 0.001	0.42938
*K. pneumoniae*	28	2129	586	32.8% (24.6–42.2)	322.557	91.629	< 0.001	0.96848
**Asia**								
*E. coli*	72	37,812	11,900	35.7% (31.2–40.5)	4138.772	98.285	< 0.001	0.38421
*K. pneumoniae*	72	20,673	5844	30.3% (26.6–34.3)	1841.097	96.198	< 0.001	0.88163
**Europe**								
*E. coli*	9	16,485	14,916	23.1% (13.9–35.9)	1109.543	99.189	< 0.001	0.53125
*K. pneumoniae*	9	7072	2833	25.4% (11.0–48.2)	1322.463	99.319	< 0.001	0.32518
**North America**								
*E. coli*	7	5862	2229	28.1% (8.9–60.8)	2116.863	99.669	< 0.001	0.50000
* K. pneumoniae*	7	3464	6657	25.0% (11.1–47.1)	550.010	98.727	< 0.001	0.45790

**Table 2:** Subgroup analysis of ESBL-producing *Escherichia coli* and *Klebsiella pneumoniae* in animals

Risk factors	Number of studies	Pooled estimates			Measure of heterogeneity			Publication bias	
		Sample size	ESBL positive	I^2^ (95%CI)	Q Value	I^2^	*Q*	Beg and Mazumdar rank *P*-value	
**Overall Animal**									
*E. coli*	17	3282	872	33.5% (13.1–36.1)	606.921	97.364	< 0.001	0.65046	
* K. pneumoniae*	16	2753	324	19.4% (9.7–34.9)	307.376	95.120	< 0.001	0.47130	
**Study year**									
**2011–2020**									
*E. coli*	11	664	346	75% (41.5–62.1)	357.913	97.206	< 0.001	0.31151	
* K. pneumoniae*	10	398	90	17.1% (6.5–37.9)	210.352	95.721	< 0.001	0.42083	
**2021–2022**									
*E. coli*	6	2618	526	61.8% (25.5–88.4)	234.455	97.867	< 0.001	0.57303	
* K. pneumoniae*	6	2355	144	23.7% (7.6–53.9)	95.062	94.740	< 0.001	0.57303	
**Samples**									
**Chickens**									
*E. coli*	5	578	287	48.0% (14.0–80.0)	209.184	98.088	< 0.001	0.14164	
*K. pneumoniae*	5	288	82	43.5% (15.0–77.0)	68.954	94.199	< 0.001	0.5000	
**Countries**									
**Nigeria**									
*E. coli*	3	442	169	33.4% (3.5–87.2)	166.522	98.799	< 0.001	0.11719	
* K. pneumoniae*	3	248	28	11.9% (8.0–17.5)	2	18.847	< 0.001	0.11719	
**Continent**									
**Africa**									
*E. coli*	9	1085	555	49.6% (28.2–71.1)	282.725	97.171	< 0.001	0.40425	
*K. pneumoniae*	9	734	166	32.9% (16.6–56.6)	139.268	94.256	< 0.001	0.40425	
**Asia**									
*E. coli*	5	341	159	44.5% (24.5–66.5)	47.327	91.548	< 0.001	1.0000	
* K. pneumoniae*	4	163	60	33.7% (14.7–60.1)	24.560	87.785	< 0.001	1.0000	
**Europe**									
*E. coli*	2	1407	62	–	–	–	–	–	
*K. pneumoniae*	2	1407	5	–	–	–	–	–	
**North America**									
*E. coli*	1	449	96	–	–	–	–	–	
*K. pneumoniae*	1	449	3	–	–	–	–	–	

**Table 3:** Subgroup analysis of ESBL-producing *Escherichia coli* and *Klebsiella pneumoniae* in the environment

Risk factors	Number of studies	Pooled estimates				Measure of heterogeneity			Publication bias	
		Sample size	ESBL positive	I^2^ (95%CI)	Q Value		I^2^	*Q*	Beg and Mazumdar rank *P*-value	
**Overall Environment**										
*E. coli*	5	1151	538	56.9% (31.6–79.0)	24.885		83.926	< 0,001	0.62421	
* K. pneumoniae*	5	1146	103	24.2% (8.2–53.4)	37.460		89.322	< 0,001	0.32719	
**Study year**										
**2011–2020**										
*E. coli*	3	1124	530	75.6% (32.8–95.1)	21.726		90.794	< 0,001	0.60151	
* K. pneumoniae*	3	1123	91	11% (5.4–21.1)	4.443		54.989	< 0,001	0.11719	
**2021–2022**										
*E. coli*	2	27	8	–	–		–	–	–	
*K. pneumoniae*	2	23	12	–	–		–	–	–	
**Continent**										
**Asia**										
*E. coli*	3	341	159	47.9% (30.2–66.1)	3.868		48.292	< 0,001	0.60151	
* K. pneumoniae*	3	163	60	28.8% (4.2–78.6)	32.240		93.796	< 0,001	0.60151	

### Prevalence of ESBL in humans

**Fig. 2:**
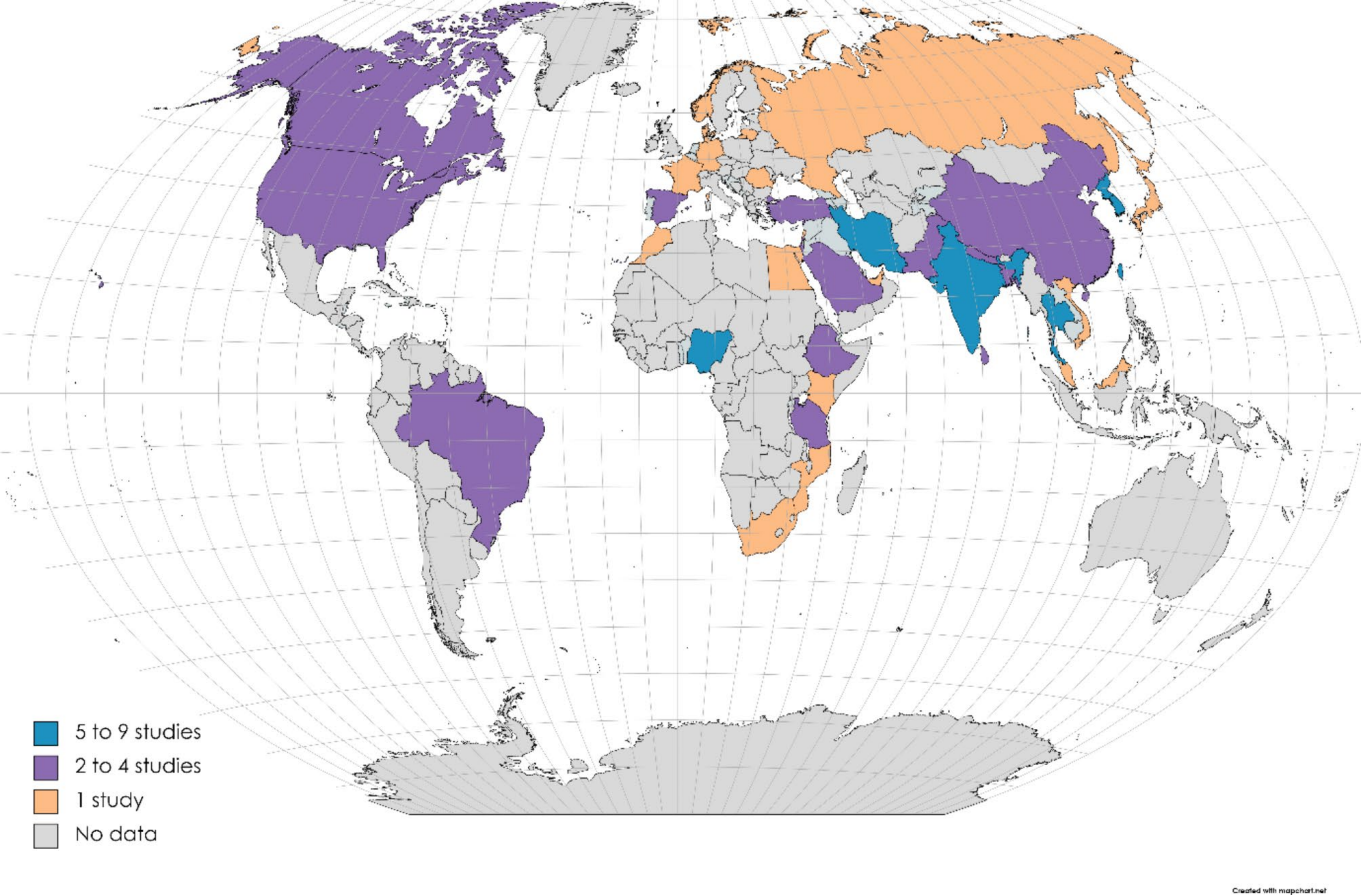
World map showing number of articles from different countries that reported the ESBL-producing *E. coli* and *K. pneumoniae* in humans (https://www.mapchart.net/world.html (accessed on 10 March 2023)

**Fig. 3:**
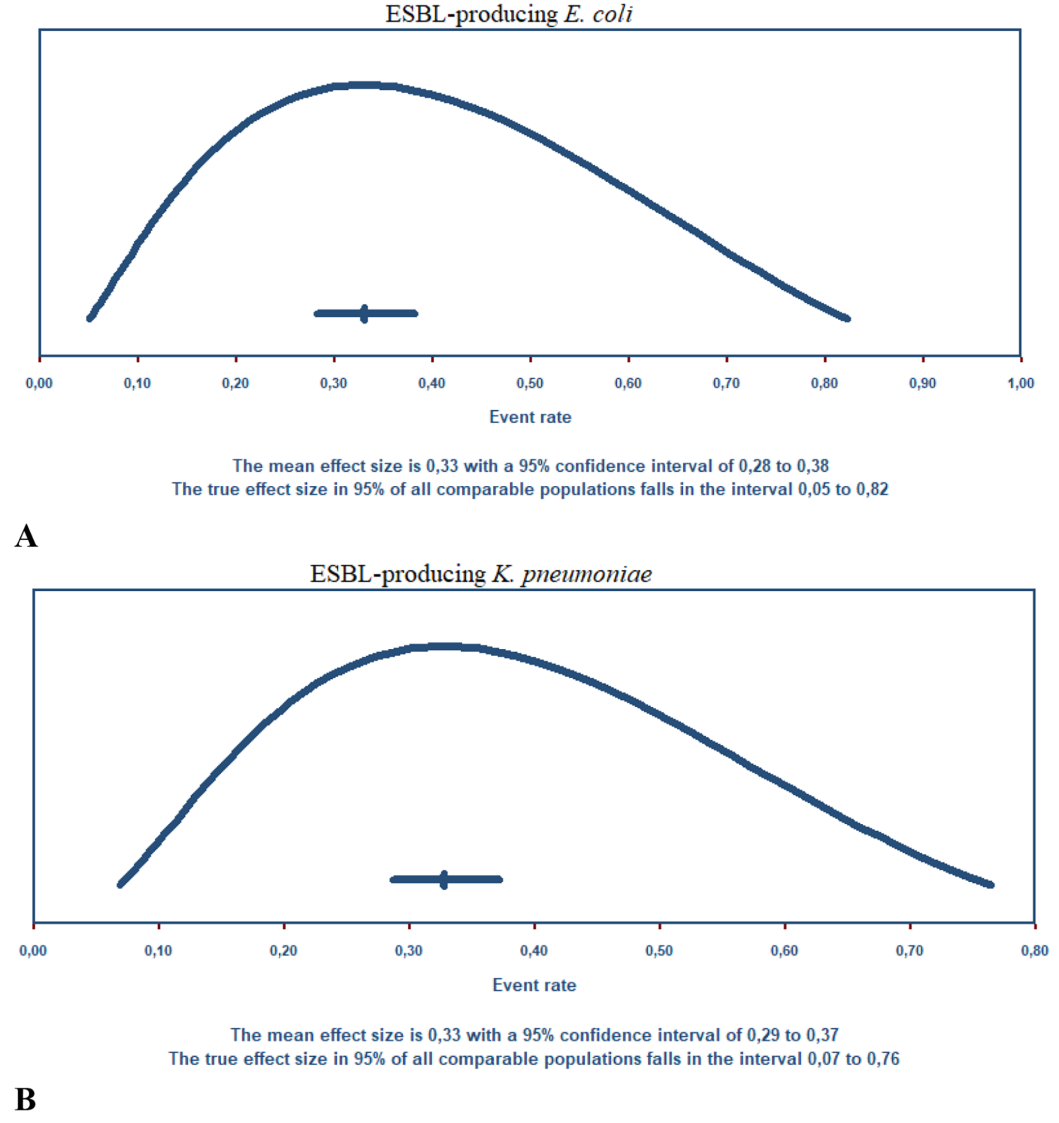
Plot of true effects: **A**) *E. coli*, the mean effect size is 0,330, the mean score is 0.82, and **B**) *K. pneumoniae*, the mean effect size is 0,327, and the mean score is 0.76

The global prevalence of co-existing ESBL-producing *E. coli* and *K. pneumoniae* in humans was investigated in 101 studies (Supplementary Table S1; Fig. 2), of which the pooled prevalence was 33.0% (0.330; 95% CI: 0.282–0.381) for *E. coli* and 32.7% (0.327; 95% CI: 0.286–0.371) for *K. pneumoniae*, ranging from 0.6 to 99.9%. The mean effect size is 0.330 with a 95% confidence interval of 0.282 to 0.381 for *E. coli* (Fig. 3A); the prediction interval (0.282 to 0.381) reflects the endpoints of this distribution. While the mean effect size is 0,327 with a 95% confidence interval of 0.286 to 0.371 for *K. pneumoniae* (Fig. 3B), the prediction interval shows that there were some populations where the effect was as low as 0.286 and others where it was as high as 0.371. According to the study year, ESBL-producing *E. coli* was more prevalent in the 2021–2022 year interval with a PPE of 53% (95% CI: 41.1–65.5%), while ESBL-producing *K. pneumoniae* were more prevalent in the 2000–2010 period with PPE of 39.7% (95% CI: 31.8–48.1). The period of year interval 1990–2000 had the lowest PPE for ESBL-producing *E. coli* and *K. pneumoniae* with 10.9% (95% CI: 04.4–24.5) and 25.4% (95% CI: 7.1–60.0), respectively.

According to settings, hospitals had a high PPE for both ESBL-producing *E. coli* and *K. pneumoniae*, with 49% (95% CI: 41.1–57.4) and 45.0% (95% CI: 38.8–53.4), respectively. The lowest PPE for ESBL-producing *E. coli* was observed in tertiary hospitals with 37.6%, and 35.1% for ESBL-producing *K. pneumoniae*. In military hospitals, *E. coli* had 42.3% and *K. pneumoniae* had 19.9% PPE. Furthermore, for the type of samples screened, ESBL-producing *E. coli* and *K. pneumoniae* were more prevalent in faecal samples with 52.7% (95% CI: 29.3–74.9) and 37.7% (95% CI: 28.7–47.7) for urine. For various specimens, *E. coli* had 34.2% PPE and *K. pneumoniae* had 28.8% PPE.

At continent level, Africa reported the highest PPE at 44.3% (95% CI: 32.2–57.2) and 32.8% (95% CI: 24.6–42.2) for ESBL-producing *E. coli* and *K. pneumoniae*. Lastly, the PPE at country level indicates that Thailand registered the highest PPE at 52.1% for ESBL-producing *E. coli*, while the USA had a PPE of 55.7% for ESBL-producing *K. pneumoniae*.

### Prevalence of ESBL in animals

The characteristics of all eligible animal studies included in this review were presented in Supplementary Table S2. Using a random-effect model, the pooled prevalence of ESBL-producing *E. coli* and *K. pneumoniae* was estimated at 33.5% (95% CI: 0.131–0.361) for *E. coli* and 19.4% (95% CI: 0.097–0.349) with high heterogeneity (I^2^: 97.364% and I^2^: 95.120%, respectively), with a mean effect size of 0.417 and 0.194 for *E. coli* and *K. pneumoniae*, respectively.

**Fig. 4:**
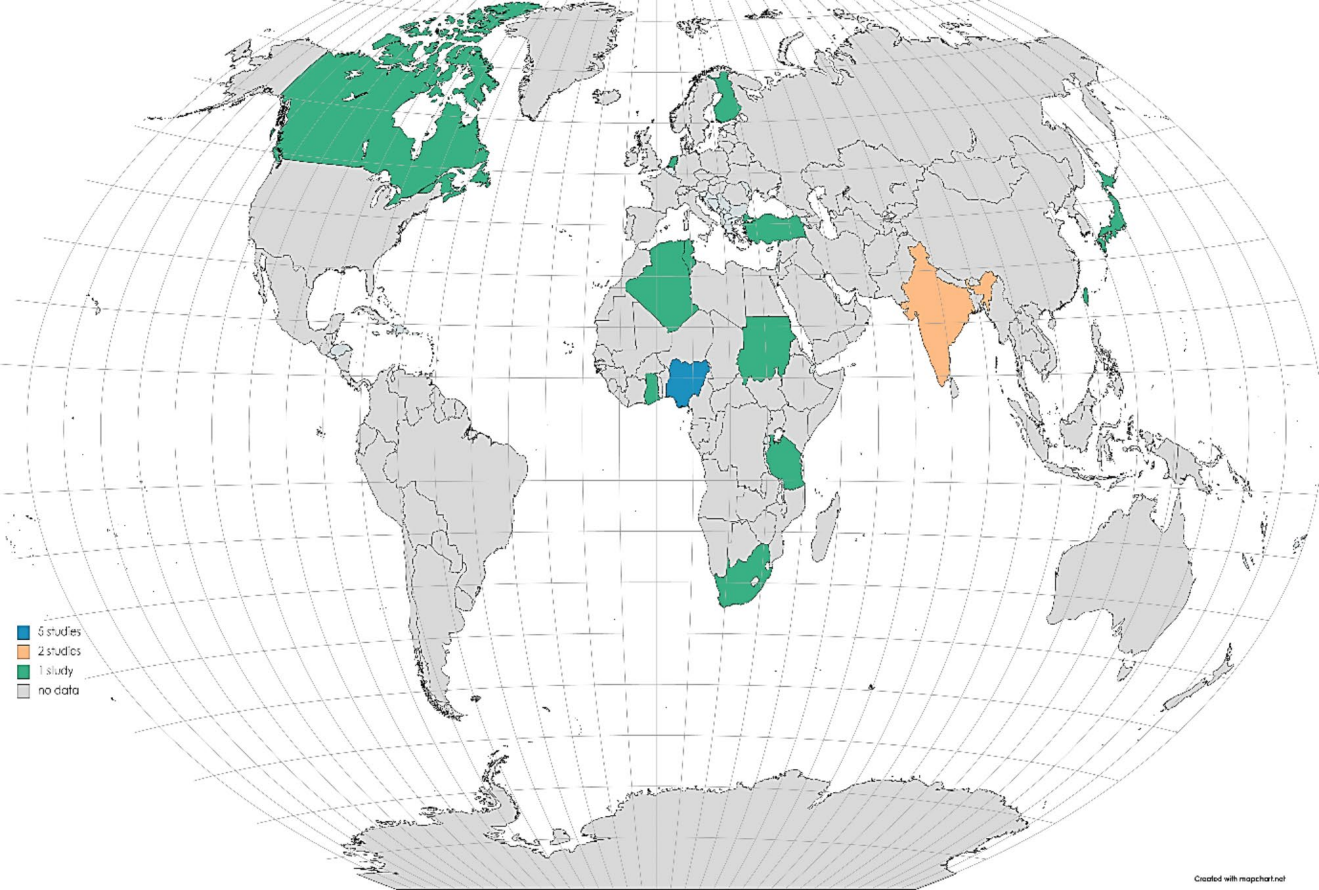
World map showing number of articles from different countries that reported the ESBL-producing *E. coli* and *K. pneumoniae* in animals (https://www.mapchart.net/world.html (accessed on 10 March 2023)

In terms of regions, the majority of the studies have performed in Africa (n = 9) (Fig. 4). For ESBL-producing *E. coli*, the highest estimate of 49.6% (95% CI: 28.2–71.1) was observed from Africa, and pooled prevalence was estimated at 49.6% (95% CI: 28.2–71.1) for ESBL-producing *K. pneumoniae*. However, Europe and North America were not included in the meta-analysis due to their low number of studies (fewer than three studies).

**Fig. 5:**
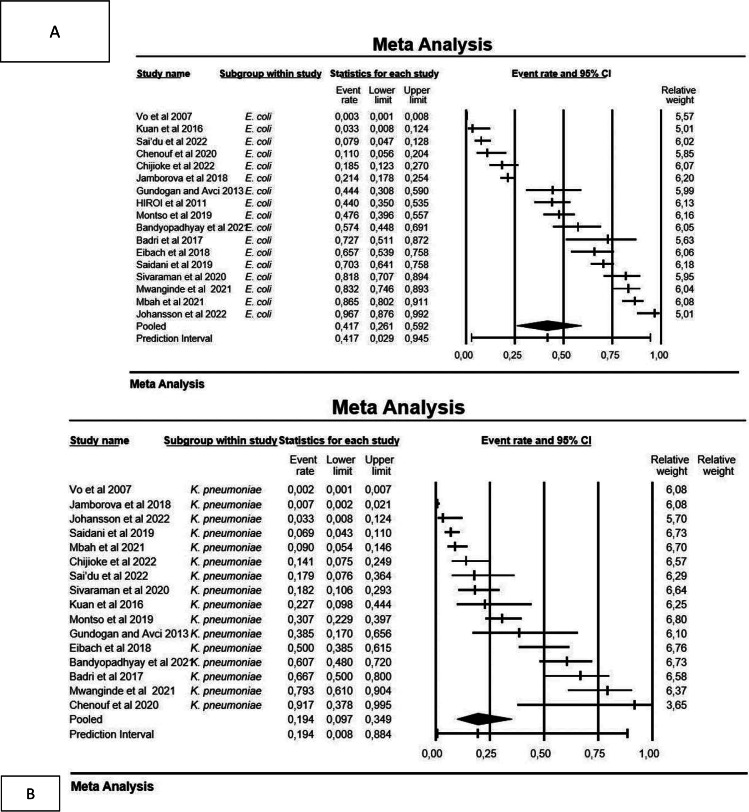
Forest plot showing the pooled estimates of (**a**) ESBL-producing *E. coli* and (**b**) ESBL-producing *K. pneumoniae* from animals’ samples. The squares demonstrate the individual point estimate. The diamond at the base indicates the pooled estimates from the overall studies

When analysing prevalence data in subgroups categorized by the countries, studies from Nigeria had a PPE of 33.4% (95% CI: 3.5–87.2) for ESBL-producing *E. coli*, while ESBL-producing *K. pneumoniae* had the lowest PPE at 11.9% (95% CI: 8.0–17.5). Furthermore, chicken sample analysis revealed a PPE of 48.0% (95% CI: 14.0–80.0) for ESBL-producing *E. coli* and 43.5% (15.0–77.0) for ESBL-producing *K. pneumoniae* observed from n = 287 and n = 82, respectively. Regarding the prevalence of ESBL-producing *E. coli* and *K. pneumoniae* according to study year, ESBL-producing *E. coli* was more prevalent in the 2011–2020 period with a PPE of 75% (95% CI: 41.5–62.1), while ESBL-producing *K. pneumoniae s*howed a PPE of 23.7% (95% CI: 7.6–53.9) in the 2001–2010 period. Figure 5 shows a forest plot of individual point estimates for the combined prevalence estimates of (A) ESBL-producing *E. coli* and (B) *K. pneumoniae* in animals.

### Prevalence of ESBL from the environment

For the environment, studies on the co-existing ESBL-producing *E. coli* and *K. pneumoniae* were conducted in five countries (Nigeria, India, Tunisia, Nepal, and Thailand) (Supplementary Table S3). The PPE for isolates from the environment were 56.9% (95% CI: 0.316–0.790), predicted interval (0.031 to 0.982) for *E. coli* and 24.2% (0.242; 95% CI: 0.082–0.534), predicted interval (0.003 to 0.973) for *K. pneumoniae*. The PPE of ESBL-producing *E. coli* in Asia was 47.9% (95% CI: 30.2–66.1), while ESBL-producing *K. pneumoniae* gave the highest estimate at 28.8% (95% CI: 4.2–78.6). The prevalence of ESBL-producing *E. coli* from 2011 to 2022 was 56.9% 95% CI: 31.6–79.0), while ESBL-producing *K. pneumoniae* had the lowest estimate at 24.2% 95% CI: 8.2–53.4).

### Prevalence of ESBL from both animals and the environment

The *E. coli* and *K. pneumoniae* were screened from 625 isolates of both animals and the environment, 96 of which were ESBL-producing *E. coli*, and 33 were ESBL-producing *K. pneumoniae* (Supplementary Table S4). The overall PPE for ESBL-producing *E. coli* was 26.8% based on three studies (95% CI: 4.9–72.3). The ESBL-producing *K. pneumoniae* had a PPE of 6.7% (95% CI: 1.5–25.5) (Table 4).

**Table 4:** Subgroup analysis of ESBL-producing *Escherichia coli* and *Klebsiella pneumoniae* in animal and the environment

Risk factors	Number of studies	Pooled estimates				Measure of heterogeneity			Publication bias	
		Sample size	ESBL positive	I^2^ (95%CI)	Q Value		I^2^	*Q*	Beg and Mazumdar rank *P*-value	
Overall animal/environment										
*E. coli*	3	625	96	26.8% (4.9–72.3)	96.018		97.917	< 0,001	0.60151	
* K. pneumoniae*	3	625	33	6.7% (1.5–25.5)	33.812		94.085	< 0,001	0.60151	

### Subgroup analysis by ESBL-resistant genes

We employed a random-effects model to analyze subgroups of ESBL genes evaluated in three or more studies. The *bla*_*SHV*_*bla*_*CTX−M*,_*bla*_*OXA*_ and *bla*_*TEM*_ genes were included in the meta-analysis for animals, humans, and the environment. As a result of the small number of studies, *bla*_*NDM*_, *bla*_*KPC*_, *bla*_*CMY*_, *bla*_*OXA*_, *bla*_*VEB*_, *bla*_*PER*_, *bla*_*GES*_, *bla*_*MOX*_, *bla*_*AmpC*_, and *bla*_*PSE*_ were not included in the meta-analysis.

### Pooled prevalence rate in humans

The overall incidence rate of ESBL-producing *E. coli* genes was 35.9% (26.0–47.1), 32.3% (22.8–43.5), 31.7% (23.0–42.0), and 2.6% (8.3–42.6), for the *bla*_*CTX−M*_,*bla*_*TEM*_,*bla*_*SHV*_ and *bla*_*OXA*_, respectively. The PPE according to ESBL-producing *K. pneumoniae* resistance genes was 35.9% (26.0–47.1), 32.3% (22.8–43.5), 31.7% (23.0–42.0) and 2.6% (8.3–42.6), for the *bla*_*CTXM*_,*bla*_*TEM*_,*bla*_*SHV*_ and *bla*_*OXA*_, respectively.

### Pooled prevalence rate in animals

ESBL-producing *E. coli* and *K. pneumoniae* in animals consisted of the *bla*_*SHV*_,*bla*_*CTXM*_,*bla*_*OXA*_, and *bla*_*TEM*_ genes. In total, four genes or gene groups were analyzed. The PPE was observed on ESBL-producing *E. coli* for the *bla*_*SHV*_,*bla*_*CTXM*_,*bla*_*OXA*_ and *bla*_*TEM*_ at 29.8% (17.0–46.8), 27.5% (14.8–45.3), 20.4% (4.7–56.9) and 19.1% (6.2–45.8), respectively. For ESBL-producing *K. pneumoniae* prevalence estimates are close to 29.7% (11.3–47.8), 26.5% (12.7–47.1), 21.3% (9.3–41.7) and 18.2% (6.9–40.1), *bla*_*OXA*_,*bla*_*TEM*_,*bla*_*SHV*_ and *bla*_*CTXM*_ respectively.

#### Pooled prevalence rate in the environment

The PPE for the *bla*_*CTXM*_ gene was estimated to be 51.4% (25.8–76.2) and 22.6% (6.5–55.2) for ESBL-producing *E. coli* and *K. pneumoniae*, respectively. For studies on the *bla*_*SHV*_ gene, PPE was estimated to be 53.2% (12.8–89.8) for ESBL-producing *E. coli* and 32.2% (8.2–71.5) for ESBL-producing *K. pneumoniae*.

### Risk of publication bias of included studies

**Fig. 6:**
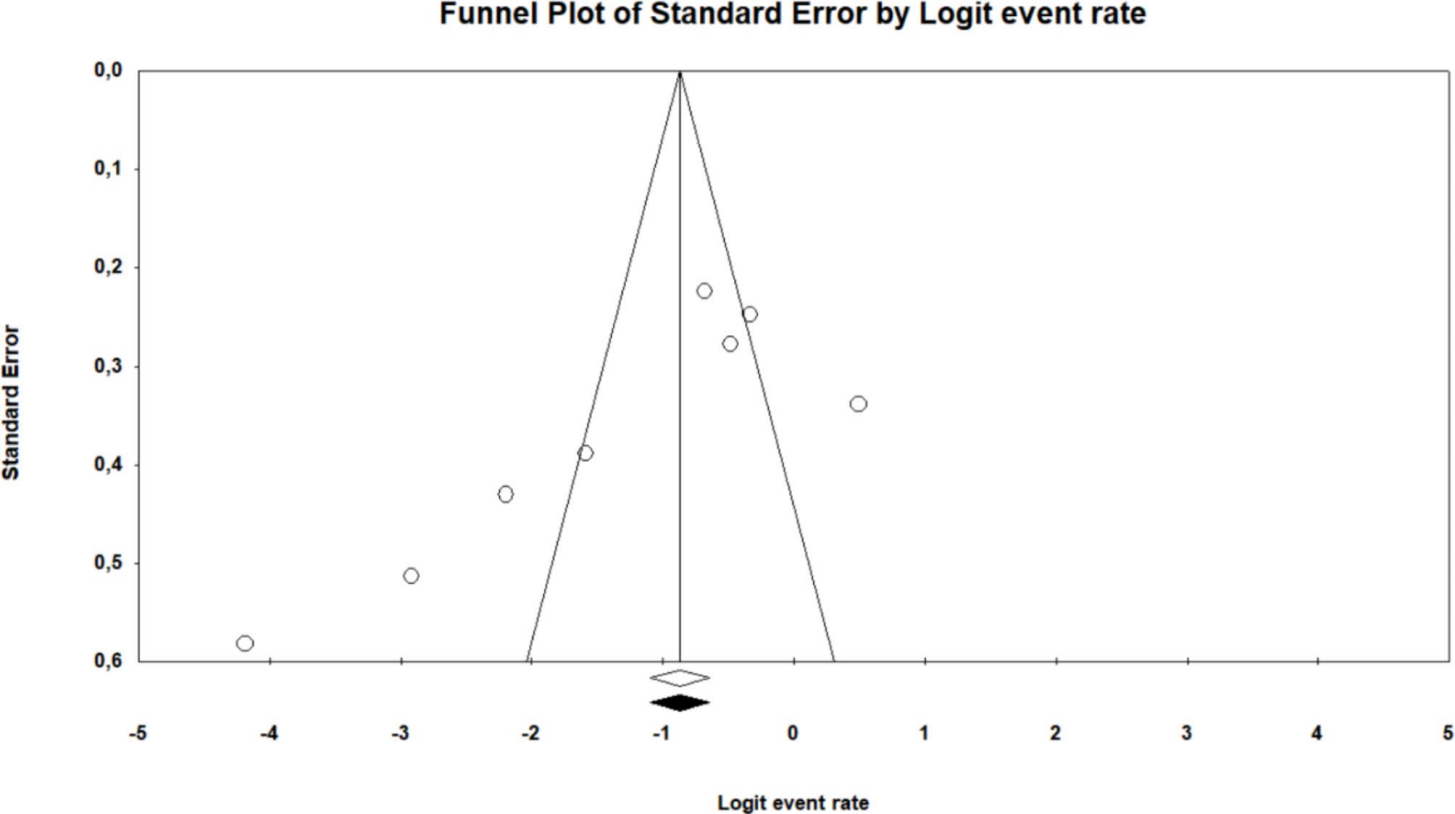
Funnel plot showing the evidence of publication bias of the studies conducted on faecal samples from humans (ESBL-producing and *K. pneumoniae*)

Publication bias was measured using funnel plots to test for symmetry, and this was further complemented using the Begg and Mazumdar rank correlation test and Egger’s regression test. The funnel plot was used to analyze the publication bias. Figure 6 displays a practically symmetrical distribution of all included studies on both sides of the funnel plot, indicating a relatively low potential for publication bias (*p*-value < 0.02595) for faecal samples from humans (ESBL-producing *E. coli* and *K. pneumoniae*).

## Discussion

In this review of data published between January 1, 1990 and November 28, 2022, key findings have been made regarding the burden of co-existing *E. coli* and *K. pneumoniae* positively expressing ESBL which provide useful information for health professionals in a variety of fields. There are some big differences between animals, humans and the environment in the prevalence of ESBL positive *E. coli* and *K. pneumoniae*. We therefore chose to examine the prevalence of ESBL positive co-existing *E. coli* and *K. pneumoniae* from each source and do a subgroup analysis. This study analyzed data on ESBL-producing co-existing *E. coli* and *K. pneumoniae* isolated from human, animal, and environmental samples worldwide. Many articles were excluded from our systematic literature review because they only reported the prevalence *E. coli* or *K. pneumoniae* separately. However, the aim of this study was to include only studies that reported both co-existing *E. coli* and *K. pneumoniae*.

### ESBL of *E. coli* and *K. pneumoniae* isolated from humans

*Escherichia coli* and *K. pneumoniae* species are the most common cause of infections, particularly in countries with underdeveloped healthcare systems [20, 21]. The ESBL-producing *E. coli* and *K. pneumoniae* strains have been identified as prominent multidrug-resistant pathogens linked to hospital-acquired infections [16, 20, 21]. They are also known to cause several common infections in children, including gastroenteritis, urinary tract infections, septicaemia, and neonatal meningitis [16, 22]. It is also known that *K. pneumoniae* is one of the most common bacteria that causes of opportunistic healthcare-associated infections, which are made worse by its ability to produce ESBL enzymes [23, 24].

The PPE based on 17,513 ESBL positive *E. coli* and 8165 ESBL positive *K. pneumoniae* was 33% and 32.7% respectively, from 101 studies in humans. This is comparatively higher than similarly reported PPE from a review conducted in Bangladesh, where PPE of ESBL-producing *E. coli* was 17% [16], and in South America, where PPE was 2.2% [15]. The study conducted in Nepal on ESBL-producing *K. pneumoniae* had a lower PPE of 5% for ESBL-producing *K. pneumoniae* [24]. The variation could be due to the result of a difference in the time of the study, also be due to differences in geographical properties, sample categories, and types, as well as sample size and identification methods.

Furthermore, this study found that *E. coli* had a high PPE of 52.7% on stool samples from 243 positive ESBL-producing *E. coli*, while *K. pneumoniae* had a high PPE of 37.7% on urine samples. This is not surprising, as *K. pneumoniae* is one of the bacteria that cause urinary tract infections [25, 26]. However, most of the stool samples containing the *stx*-positive gene of *E. coli* were typical diarrheal samples [27].

### ESBL of *E. coli* and *K. pneumoniae* isolated from animals

In this study, 872 ESBL-producing *E. coli* isolates from 17 studies and 324 ESBL-producing *K. pneumoniae* from 16 studies were subjected to meta-analysis. The *E. coli* and *K. pneumoniae* PPE from this study are 33.5% and 19.4%, respectively. Our results are comparable to a previous systematic review study by Islam et al. [16] that reported prevalence of 22% for for ESBL-producing *E. coli* in animals. However, PEE in our study was higher than the one obtained in India, where PPE of ESBL-producing *E. coli* and *K. pneumoniae* was 9% and 10%, respectively [28]. The variation could be the result of a difference in the time of the study. The other reason could be the methodology used to detect the number of published articles included in the current study.

The subgroup analysis at continental level showed that the PPE was higher for ESBL-producing *E. coli* in Africa and Asia, with 49.6% and 44.5%, respectively. Our findings were consistent with a previous study that reported ESBL in *E. coli* isolated from environments in Bangladesh at 39% [16]. The PPE from chicken samples was 48.0% for *E. coli* and 43.5% for *K. pneumoniae*. Other animals such as cattle, goats, birds, dogs, cats, camels, fish, and horses were not included in the meta-analysis due to the low number of samples. Country-specific findings indicated that Nigeria had a PPE of 33.4% and 11.9% for *E. coli* and *K. pneumoniae*, respectively. Other countries, including Sudan [29], India [9], Algeria [30], Ghana [31], Turkey [32], Japan [33], Canada [34], Finland [35], Taiwan [36], South Africa [37], Tanzania [38], Tunisia [39], India [40], and the Netherland [41] were not included in the meta-analysis due to the low number of studies.

### ESBL of *E. coli* and *K. pneumoniae* isolated from the environment

This systematic review and meta-analysis showed that ESBL-producing *E. coli* and *K. pneumoniae* were isolated from various types of environmental samples, such as water, manure, hospital sewage, and raw vegetables. The PPE of ESBL-producing *E. coli* was 56.9% (31.6–79.0) while the PPE of ESBL-producing *K. pneumoniae* was 24.2%. This could be due to the possibility of traces of previously consumed antibiotics and antimicrobial-resistant bacteria in faecal and urine waste. This waste and wastewater can contribute to the spread of antimicrobial-resistant bacteria in the environment possibly having a negative impact on human health. Many of these species have genes for antibiotic resistance, which are eventually incorporated into genetic mobile platforms that can spread among bacterial populations in soil and water.

The PPE of ESBL-producing *E. coli* from environments was 39%, which is comparable to a previous study conducted in Bangladesh that reported a pooled prevalence of 39% for ESBL-producing *E. coli* from environments [16]. According to the literature, there is no meta-analysis on ESBL-producing *K. pneumoniae* in the environment. Most of the studies were conducted from 2011 to 2020 with a PPE of 75.6% for ESBL-producing *E. coli* and 11% for ESBL-producing *K. pneumoniae*. Data recorded from the included articles showed that the studies were from only five countries, namely Nigeria, India, Tunisia, Nepal, and Thailand.

### ESBL genes in *E. coli* and *K. pneumoniae* isolated from animals, humans, and the environment

In recent years, there has been an increase in ESBL-producing *E. coli* and *K. pneumoniae*, mostly identified using phenotypic methods [42, 43]. We report the first global PPE of co-existing ESBL-producing *E. coli* and *K. pneumoniae*. A number of studies have investigated *bla*_*TEM*_, *bla*_*SHV*_, *bla*_*OXA*_, and *bla*_*CTX−M*_ genes, with *bla*_*CTX−M*_ being the type most commonly associated with ESBL [43–45]. These genes reside on plasmids, which can be transferred horizontally to other bacteria [46].

Among the *bla*-genes screened in this study, bla_*CTX−M*_ and *bla*_*SHV*_ were the most detected from ESBL-producing *E. coli* and *K. pneumoniae* from animals, humans, and the environment. The *bla*_*TEM*_ have been reported from vegetables in Finland [47] and in southern Thailand [48]. In this review, *bla*_*CTX−M*_ was the gene most detected in animals, humans, and the environment. It has also been reported that *K. pneumoniae* and *E. coli* from dogs and cats possess *bla*_*CTX−M*_ type genes [49, 50]. Lately, *bla*_*CTX−M*_ enzymes are the most common ESBL type because they have environmental origins [51]. The *bla*_*CTX−M*_ enzyme is divided into five subgroups based on its amino acid composition: *bla*_*CTX−M−1*_, *bla*_*CTX−M−2*_, *bla*_*CTX−M−8*_, *bla*_*CTX−M−9*_, and *bla*_*CTX−M−25*_ [46]. Globally, carbapenem-resistant Enterobacteriaceae are increasing due to the growing usage of carbapenems to treat ESBL-producing infections [52].

In this systematic review and meta-analysis, we also report the presence of *bla*_*KPC*_ in humans. These findings warrant further investigation of the presence of carbapenem-resistant genes since such resistant *E. coli* and *K. pneumoniae* are among top priority pathogens on the list of World Health Organization [WHO] for the development of antimicrobials [53].

### “One Health” perspective

“One-Health” concept refers to the collaborative effort between local, national, and global disciplines for the purpose of attaining optimal health for humans, animals, and the environment [54–56]. Antimicrobial use practices in companion animals are similar to those in humans; that is, drugs are primarily administered for treating clinical infections, such as post-surgery infections, with some being used as prophylaxis- [57]. A major obstacle to addressing antimicrobial resistance has been the blame game between human medicine and the agriculture sectors regarding who is responsible for the increase in antimicrobial resistance due qof bacteria causing zoonotic diseases [58].

This study confirmed the co-existence of ESBL-producing *E. coli* and *K. pneumoniae* prevalence in animals, humans, and the environment (water, manure, hospitals sewage, and raw vegetables). Our results highlight the significance of “One Health,” as ESBL-producing *E. coli* and *K. pneumoniae* were detected in humans, animals, and the environment. Moreover, the bla_*CTX−M*_, and *bla*_*SHV*_ were detected in ESBL-producing *E. coli* and *K. pneumoniae* isolated from animals, humans, and the environment, hence the “One Health” concern. Future researchers should be encouraged to use the “One Health” approach to develop methodologies that explicitly examine interlinks between human-animal-environment frameworks, with a particular emphasis on antibiotic resistance in zoonotic diseases.

### Limitations

To assess ESBL-producing *E. coli* and *K. pneumoniae* levels globally, this study relied on peer-reviewed publications. Reviews, theses, and preprint articles that potentially include essential information on ESBL-producing *E. coli* and *K. pneumoniae* were excluded from our review as per PRISMA guidelines for writing systematic reviews and meta-analysis. There may have been articles published in other languages that were missed since the search strategy was limited to articles published in English. The pooled prevalence of some countries and continents was not calculated because there are few published studies. Some antimicrobial resistance genes were also not included in this meta-analysis due to the small number of studies. In a meta-analysis, publication bias may be evaluated using funnel plots, regressions, and Begg’s adjusted rank correlation test [59]. However, meta-analyses that include fewer than 10 studies or high amounts of heterogeneity between studies can lead to misleading results from these evaluation tools. In the presence of a high level of heterogeneity, it is very difficult to evaluate the true results of statistically significant publication bias tests. Since there is high heterogeneity across all analyses, readers should be cautious when interpreting pooled analyses and subgroups.

## Conclusion

This study is the first meta-analysis that estimates the prevalence of co-existing ESBL-producing *E*. *coli* and *K. pneumoniae* in animals, humans, and the environment worldwide. We further observed that bla_*CTX−M*_, and *bla*_*SHV*_ were the most frequently detected genes from ESBL-producing *E. coli* and *K. pneumoniae* infecting animals, humans, and the environment. This study presents robust and valuable data that can serve as a useful reference for doctors, veterinarians, and environmental scientists, as it informs them about the prevalence of co-existing ESBL-producing *E. coli* and *K. pneumoniae* recovered from humans, animals, and the environment. “One-health” surveillance is vital for source tracking and mitigating the spread of antimicrobial-resistant bacteria. ESBL-containing bacteria should be included in emerging state and national surveillance systems. Globally, alternative treatments for antimicrobial drug resistance should be well researched, planned, and implemented.

### Electronic supplementary material

Below is the link to the electronic supplementary material.

Supplementary Material 1

## Data Availability

Data will be made available on request.

## Abbreviations

PROSPERO: International Prospective Register of Systematic Reviews
PRISMA: Preferred Reporting Items for Systematic Reviews and Meta-Analysis
WHO: World Health Organization
ESBL: Extended Spectrum Beta Lactamase
PPE: Pooled prevalence estimates
CI: Confidence interval
PP: Pooled prevalence
AMR: Antimicrobial Resistance
MDR: Multi-drug resistant
JBI: Joanna Briggs Institute
