# Supplementary material for: “One Health” perspective on prevalence of co-existing extended-spectrum β-lactamase (ESBL)-producing Escherichia coli and Klebsiella pneumoniae: a comprehensive systematic review and meta-analysis
Source: Ann Clin Microbiol Antimicrob. 2023 Sep 22;22:88. doi: 10.1186/s12941-023-00638-3 (PMC10517531; doi:10.1186/s12941-023-00638-3)
Supplement: Supplementary file 1 — Supplementary Material 1 [file 12941_2023_638_MOESM1_ESM.docx]

**“One Health” perspective on prevalence of co-existing extended-spectrum *β-*lactamase (ESBL)-producing *Escherichia coli* and *Klebsiella pneumoniae*: A comprehensive systematic review and meta-analysis**

Tsepo Ramatla^a^*, Tshepo Mafokwane^b^, Kgaugelo Lekota^a^, Maropeng Monyama^b^, George Khasapane^3^, Naledi Serage^a^, Jane Nkhebenyane^c^, Carlos Bezuidenhout^a^, Oriel Thekisoe^a^

^a^Unit for Environmental Sciences and Management, North-West University, Potchefstroom, 2520, South Africa.

^a^Department of Life and Consumer Sciences, University of South Africa, Florida, 1710, South Africa.

^a^Department of Life Sciences, Central University of Technology, Bloemfontein, 9300, South Africa.

Corresponding author: Tsepo Ramatla

E-mail addresses: ra21205450@gmail.com: Tel: +27-18-299-2521

**Table S1.** The Joanna Briggs Institute (JBI) critical appraisal checklist for studies reporting prevalence data.

|  | Authors & years | 1 | 2 | 3 | 4 | 5 | 6 | 7 | 8 | 9 | Total |
| --- | --- | --- | --- | --- | --- | --- | --- | --- | --- | --- | --- |
| 1 | Bayraktar et al 2019 | 1 | 1 | 1 | 1 | 0 | 1 | 1 | 1 | 0 | 7 |
| 2 | Bazzaz et al 2009 | 1 | 1 | 1 | 1 | 0 | 1 | 1 | 1 | 1 | 8 |
| 3 | Ben-Chetrit et al 2019 | 1 | 1 | 1 | 1 | 0 | 1 | 1 | 1 | 0 | 7 |
| 4 | Bishara et al 2005 | 1 | 1 | 1 | 1 | 0 | 1 | 1 | 1 | 1 | 8 |
| 5 | Chander et al 2013 | 1 | 1 | 1 | 1 | 0 | 1 | 1 | 1 | 1 | 8 |
| 6 | Cheng et al 2014 | 1 | 1 | 1 | 1 | 1 | 1 | 1 | 1 | 0 | 8 |
| 7 | Cho et al 2015 | 1 | 1 | 1 | 1 | 1 | 1 | 1 | 1 | 1 | 9 |
| 8 | Chopra et al 2015 | 1 | 1 | 1 | 1 | 0 | 1 | 1 | 1 | 0 | 7 |
| 9 | Damiano et al 2021 | 1 | 1 | 0 | 0 | 0 | 1 | 1 | 1 | 0 | 5 |
| 10 | Diriba et al 2020 | 1 | 1 | 1 | 1 | 0 | 1 | 1 | 1 | 1 | 8 |
| 11 | Du et al 2002 | 1 | 1 | 1 | 1 | 0 | 1 | 1 | 1 | 1 | 8 |
| 12 | Fatima et al 2018 | 1 | 1 | 1 | 1 | 0 | 1 | 1 | 1 | 1 | 8 |
| 13 | Han et al 2014 | 1 | 1 | 1 | 1 | 1 | 1 | 1 | 1 | 1 | 9 |
| 14 | Han et al 2015 | 1 | 1 | 1 | 1 | 0 | 1 | 1 | 1 | 1 | 8 |
| 15 | Harris et al 2015 | 1 | 1 | 1 | 1 | 1 | 1 | 1 | 1 | 1 | 9 |
| 16 | Hyle et al 2004 | 1 | 1 | 1 | 1 | 1 | 1 | 1 | 1 | 1 | 9 |
| 17 | Jiménez-Guerra et al 2017 | 1 | 1 | 1 | 1 | 1 | 1 | 1 | 1 | 1 | 9 |
| 18 | Kader et al 2005 | 1 | 1 | 1 | 1 | 0 | 1 | 1 | 1 | 1 | 8 |
| 19 | Kammili et al 2014 | 1 | 0 | 1 | 0 | 0 | 1 | 1 | 0 | 1 | 5 |
| 20 | Kang et al 2004 | 1 | 1 | 1 | 1 | 0 | 1 | 1 | 1 | 1 | 8 |
| 21 | Karki et al 2021 | 1 | 1 | 1 | 1 | 0 | 1 | 1 | 1 | 1 | 8 |
| 22 | Kayastha et al 2020 | 1 | 1 | 1 | 1 | 1 | 1 | 1 | 1 | 1 | 9 |
| 23 | Kernéis et al 2015 | 1 | 1 | 0 | 1 | 0 | 1 | 1 | 1 | 1 | 7 |
| 24 | Kim et al 2014 | 1 | 1 | 1 | 1 | 1 | 1 | 1 | 1 | 1 | 9 |
| 25 | Lee 2007 | 1 | 1 | 1 | 1 | 0 | 1 | 1 | 1 | 1 | 8 |
| 26 | Lee wet al 2012 | 1 | 1 | 1 | 1 | 0 | 1 | 1 | 1 | 1 | 8 |
| 27 | Lemarie et al 2009 | 1 | 1 | 1 | 1 | 0 | 1 | 1 | 1 | 0 | 7 |
| 28 | Letara et al 2021 | 1 | 1 | 1 | 1 | 1 | 1 | 1 | 1 | 1 | 9 |
| 29 | Liang et al 2021 | 1 | 1 | 1 | 1 | 1 | 0 | 1 | 1 | 0 | 7 |
| 30 | Liu et al 2011 | 1 | 1 | 1 | 1 | 0 | 1 | 1 | 1 | 1 | 8 |
| 31 | Lowe et al 2012 | 1 | 1 | 1 | 1 | 0 | 1 | 1 | 1 | 1 | 8 |
| 32 | Mendelson et al 2005 | 1 | 1 | 1 | 1 | 0 | 1 | 1 | 1 | 1 | 8 |
| 33 | Ozgunes et al 2006 | 1 | 1 | 1 | 1 | 0 | 1 | 1 | 1 | 1 | 8 |
| 34 | Quirante et al 2011 | 1 | 1 | 1 | 1 | 0 | 1 | 1 | 1 | 0 | 7 |
| 35 | Rahman et al 2004 | 1 | 1 | 1 | 1 | 0 | 1 | 1 | 1 | 0 | 7 |
| 36 | Samaha-Kfoury et al 2005 | 1 | 1 | 1 | 1 | 0 | 1 | 1 | 0 | 0 | 6 |
| 37 | Sawatwong et al 2019 | 1 | 1 | 1 | 1 | 0 | 1 | 1 | 1 | 0 | 7 |
| 38 | Shaikh et al 2014 | 1 | 1 | 1 | 1 | 0 | 1 | 1 | 1 | 1 | 8 |
| 39 | Shaikh et al 2010 | 1 | 1 | 1 | 1 | 0 | 1 | 1 | 1 | 1 | 8 |
| 40 | Shanthi et al 2010 | 1 | 1 | 0 | 1 | 0 | 1 | 1 | 0 | 1 | 6 |
| 41 | Sharif et al 2016 | 1 | 1 | 1 | 1 | 0 | 1 | 1 | 1 | 1 | 8 |
| 42 | Superti et al 2009 | 1 | 1 | 1 | 1 | 0 | 1 | 1 | 1 | 1 | 8 |
| 43 | Taneja et al 2010 | 1 | 1 | 1 | 1 | 0 | 1 | 1 | 0 | 1 | 7 |
| 44 | Tola et al 2021 | 1 | 1 | 1 | 1 | 0 | 1 | 1 | 1 | 1 | 8 |
| 45 | Tulara, 2018 | 1 | 1 | 1 | 1 | 0 | 1 | 1 | 0 | 1 | 7 |
| 46 | Vargas et a 2009 | 1 | 1 | 1 | 1 | 0 | 1 | 1 | 1 | 1 | 8 |
| 47 | Viet et al 2021 | 1 | 1 | 1 | 1 | 0 | 1 | 1 | 1 | 1 | 8 |
| 48 | Wang et al 2011 | 1 | 1 | 1 | 1 | 0 | 1 | 1 | 1 | 1 | 8 |
| 49 | Wang et al 2019 | 1 | 1 | 1 | 1 | 0 | 1 | 1 | 1 | 1 | 8 |
| 50 | Yang et al 2010 | 1 | 1 | 0 | 1 | 0 | 1 | 1 | 1 | 0 | 6 |
| 51 | Yilmaz et al 2008 | 1 | 1 | 0 | 1 | 0 | 1 | 1 | 1 | 1 | 7 |
| 52 | Yousef et al 2016 | 1 | 1 | 1 | 1 | 0 | 1 | 1 | 1 | 1 | 8 |
| 53 | Dissanayake et al 2012 | 1 | 1 | 1 | 0 | 0 | 1 | 1 | 0 | 1 | 6 |
| 54 | Leistner et al 2014 | 1 | 1 | 1 | 1 | 1 | 1 | 1 | 1 | 1 | 9 |
| 55 | Abrar et al 2017 | 1 | 1 | 1 | 1 | 0 | 1 | 1 | 0 | 1 | 7 |
| 56 | Alfaresi et al 2011 | 1 | 1 | 1 | 1 | 0 | 1 | 1 | 0 | 0 | 6 |
| 57 | Barguigua et al 2011 | 1 | 1 | 1 | 1 | 0 | 1 | 1 | 0 | 1 | 7 |
| 58 | Baroud et al 2013 | 1 | 1 | 1 | 0 | 0 | 1 | 1 | 0 | 0 | 5 |
| 59 | Doi et al 2012 | 1 | 1 | 1 | 1 | 0 | 1 | 1 | 0 | 1 | 7 |
| 60 | Edelstein et al 2003 | 1 | 1 | 1 | 1 | 0 | 1 | 1 | 0 | 1 | 7 |
| 61 | Kim et al 2002 | 1 | 1 | 1 | 1 | 1 | 1 | 1 | 1 | 1 | 9 |
| 62 | Kiratisin et al 2008 | 1 | 1 | 1 | 1 | 0 | 1 | 1 | 0 | 1 | 7 |
| 63 | Kizilates et al 2021 | 1 | 1 | 0 | 1 | 0 | 1 | 1 | 1 | 1 | 7 |
| 64 | Krishnamurthy et al 2013 | 1 | 1 | 1 | 1 | 0 | 1 | 1 | 1 | 1 | 8 |
| 65 | Lee et al 2007 | 1 | 1 | 1 | 1 | 1 | 1 | 1 | 1 | 1 | 9 |
| 66 | Nakama et al 2016 | 1 | 1 | 1 | 1 | 1 | 1 | 1 | 1 | 1 | 9 |
| 67 | Olalekan et al 2019 | 1 | 1 | 1 | 1 | 0 | 1 | 1 | 1 | 1 | 8 |
| 68 | Pornsinchai et al 2015 | 1 | 1 | 1 | 1 | 1 | 1 | 1 | 1 | 1 | 9 |
| 69 | Priyadharshana et al 2019 | 1 | 1 | 1 | 1 | 1 | 1 | 1 | 1 | 1 | 9 |
| 70 | Seputiene et al 2010 | 1 | 1 | 1 | 1 | 1 | 1 | 1 | 1 | 1 | 9 |
| 71 | SIU et al 1999 | 1 | 1 | 0 | 1 | 1 | 1 | 1 | 0 | 1 | 7 |
| 72 | Thirapanmethee et al 2014 | 1 | 1 | 1 | 1 | 0 | 1 | 1 | 0 | 1 | 7 |
| 73 | Tofteland et al 2007 | 1 | 1 | 1 | 1 | 0 | 1 | 1 | 0 | 1 | 7 |
| 74 | Zerr et al 2016 | 1 | 1 | 1 | 1 | 0 | 1 | 1 | 1 | 1 | 8 |
| 75 | ABE et al 2021 | 1 | 1 | 0 | 0 | 0 | 1 | 1 | 0 | 1 | 5 |
| 76 | Amer et al 201 | 1 | 1 | 1 | 0 | 1 | 1 | 1 | 0 | 1 | 7 |
| 77 | Chirindzeet al 2018 | 1 | 1 | 1 | 0 | 0 | 1 | 1 | 0 | 1 | 6 |
| 78 | Deji-Agboolaet al 2020 | 1 | 1 | 1 | 1 | 1 | 1 | 1 | 1 | 1 | 9 |
| 79 | Ghenea et al 2022 | 1 | 1 | 0 | 0 | 0 | 1 | 1 | 1 | 1 | 6 |
| 80 | Hansen et al 2012 | 1 | 1 | 0 | 1 | 0 | 1 | 1 | 1 | 1 | 7 |
| 81 | Hasani et al 2017 | 1 | 1 | 1 | 1 | 1 | 1 | 1 | 1 | 1 | 9 |
| 82 | Jahromy et al 2019 | 1 | 1 | 1 | 1 | 1 | 1 | 1 | 0 | 1 | 8 |
| 83 | Juma et al 2016 | 1 | 1 | 1 | 1 | 0 | 1 | 1 | 0 | 1 | 7 |
| 84 | Karim et al 2017 | 1 | 1 | 0 | 1 | 0 | 1 | 1 | 0 | 1 | 6 |
| 85 | Khanal et al 2022 | 1 | 1 | 1 | 1 | 0 | 1 | 1 | 0 | 1 | 7 |
| 86 | Kim et al 2005 | 1 | 1 | 1 | 1 | 0 | 1 | 1 | 0 | 1 | 7 |
| 87 | Mulvey et al 2004 | 1 | 1 | 1 | 1 | 0 | 1 | 1 | 0 | 1 | 7 |
| 88 | Ogbolu et al 2013 | 1 | 1 | 1 | 1 | 0 | 1 | 1 | 1 | 1 | 8 |
| 89 | Riaz et al 2015 | 1 | 1 | 1 | 1 | 0 | 1 | 1 | 0 | 1 | 7 |
| 90 | Somily et al 2015 | 1 | 1 | 0 | 1 | 0 | 1 | 1 | 0 | 1 | 6 |
| 91 | Ushie et al 2020 | 1 | 1 | 0 | 1 | 0 | 1 | 1 | 0 | 1 | 6 |
| 92 | Yarima et al 2020 | 1 | 1 | 1 | 1 | 0 | 1 | 1 | 1 | 1 | 8 |
| 93 | Adeyankinnu et al 2014 | 1 | 1 | 1 | 1 | 1 | 1 | 1 | 1 | 1 | 9 |
| 94 | Ambrose et al 2003 | 1 | 1 | 1 | 1 | 0 | 1 | 1 | 0 | 1 | 7 |
| 95 | Arbab et al 2012 | 1 | 1 | 1 | 1 | 0 | 1 | 1 | 0 | 1 | 7 |
| 96 | Aziz et al 2019 | 1 | 1 | 1 | 1 | 0 | 1 | 1 | 0 | 0 | 6 |
| 97 | Mahomed and Coovadia, 2015 | 1 | 1 | 1 | 1 | 0 | 1 | 1 | 0 | 1 | 7 |
| 98 | Mofolorunsho et al 2021 | 1 | 1 | 1 | 1 | 0 | 1 | 1 | 0 | 1 | 7 |
| 99 | Ngoi et al 2021 | 1 | 1 | 1 | 1 | 0 | 1 | 1 | 0 | 1 | 7 |
| 100 | Onanuga et al 2019 | 1 | 1 | 1 | 1 | 0 | 1 | 1 | 1 | 1 | 8 |
| 101 | Siriphap et al 2022 | 1 | 1 | 1 | 1 | 0 | 1 | 1 | 1 | 0 | 7 |
| 102 | Badri et al 2017 | 1 | 1 | 1 | 1 | 0 | 1 | 1 | 0 | 0 | 6 |
| 103 | Bandyopadhyay et al 2021 | 1 | 1 | 1 | 1 | 0 | 1 | 1 | 0 | 0 | 6 |
| 104 | Chenouf et al 2020 | 1 | 1 | 1 | 1 | 0 | 1 | 1 | 0 | 0 | 6 |
| 105 | Chijioke et al 2022 | 1 | 1 | 1 | 1 | 1 | 1 | 1 | 1 | 0 | 8 |
| 106 | Eibach et al 2018 | 1 | 1 | 1 | 1 | 0 | 1 | 1 | 1 | 0 | 7 |
| 107 | Gundogan and Avci 2013 | 1 | 1 | 0 | 1 | 0 | 1 | 1 | 0 | 0 | 5 |
| 108 | Hiroi et al 2011 | 1 | 1 | 0 | 1 | 0 | 1 | 1 | 0 | 0 | 5 |
| 109 | Jamborova et al 2018 | 1 | 1 | 1 | 1 | 0 | 1 | 1 | 1 | 0 | 7 |
| 110 | Johansson et al 2022 | 1 | 1 | 0 | 1 | 0 | 1 | 1 | 0 | 0 | 5 |
| 111 | Kuan et al 2016 | 1 | 1 | 0 | 1 | 0 | 1 | 1 | 0 | 0 | 5 |
| 112 | Mbah et al 2021 | 1 | 1 | 1 | 1 | 0 | 1 | 1 | 0 | 0 | 6 |
| 113 | Montso et al 2019 | 1 | 1 | 1 | 1 | 0 | 1 | 1 | 1 | 0 | 7 |
| 114 | Mwanginde et al 2021 | 1 | 1 | 1 | 1 | 0 | 1 | 1 | 1 | 0 | 7 |
| 115 | Sai’du et al 2022 | 1 | 1 | 1 | 1 | 1 | 1 | 1 | 1 | 0 | 8 |
| 116 | Saidani et al 2019 | 1 | 1 | 1 | 1 | 0 | 1 | 1 | 0 | 0 | 6 |
| 117 | Sivaraman et al 2020 | 1 | 1 | 1 | 1 | 0 | 1 | 1 | 0 | 0 | 6 |
| 118 | Vo et al 2007 | 1 | 1 | 1 | 1 | 0 | 1 | 1 | 0 | 0 | 6 |
| 119 | Atta et al 2022 | 1 | 1 | 1 | 1 | 0 | 1 | 1 | 0 | 0 | 6 |
| 120 | Devi et al 2020 | 1 | 1 | 1 | 1 | 0 | 1 | 1 | 1 | 0 | 7 |
| 121 | Hassen et al 2020 | 1 | 1 | 1 | 1 | 0 | 1 | 1 | 0 | 0 | 6 |
| 122 | Mahato et al 2019 | 1 | 1 | 0 | 1 | 0 | 1 | 1 | 1 | 0 | 6 |
| 123 | Romyasamit et al 2021 | 1 | 1 | 1 | 1 | 0 | 1 | 1 | 0 | 0 | 6 |
| 124 | Sivaraman et al 2021 | 1 | 1 | 1 | 1 | 0 | 1 | 1 | 0 | 0 | 6 |
| 125 | Soré et al 2021 | 1 | 1 | 1 | 1 | 0 | 1 | 1 | 1 | 0 | 7 |
| 126 | Kurittu et al 2021 | 1 | 1 | 1 | 1 | 0 | 1 | 1 | 1 | 0 | 7 |

**Table S1.** List and characteristics of eligible studies included in the meta-analysis with respect to ESBL-producing *Escherichia coli* and *Klebsiella pneumoniae* in human.

|  | **Authors & years** | **Country** | **Samples type** | **ESBL diagnostic Methods** | | ***E. coli*** | | | | | ***K. pneumoniae*** | | | |
| --- | --- | --- | --- | --- | --- | --- | --- | --- | --- | --- | --- | --- | --- | --- |
|  |  |  |  |  |  | **Isolates** | | ***ESBL***  ***+*** | | | **Isolates** | | | ***ESBL***  ***+*** |
| 1 | Bayraktar et al 2019 | Turkey | Blood | CLSI and E-test | | 632 | | 224 | | | 439 | | | 137 |
| 2 | Bazzaz et al 2009 | Iran | Urine, blood, wound swab, vaginal swab, urethral discharge, eye swab, plural fluids, and cerebral spinal fluid | CLSI, | | 106 | | 61 | | | 100 | | | 61 |
| 3 | Ben-Chetrit et al 2019 | Israel | Blood | CLSI | | 48 | | 12 | | | 32 | | | 14 |
| 4 | Bishara et al 2005 | Israel | Blood and urine | CLSI | | 547 | | 57 | | | 765 | | | 241 |
| 5 | Chander et al 2013 | Nepal | Urine | CLSI and PCDDT | | 444 | | 60 | | | 145 | | | 24 |
| 6 | Cheng et al 2014 | Taiwan | Blood and Sputum | CLSI and VITEK | | 473 | | 228 | | | 473 | | | 245 |
| 7 | Cho et al 2015 | Republic of Korea | Urine | CLSI, VITEK and semi-automated system, MicroScan | | 277 | | 217 | | | 277 | | | 60 |
| 8 | Chopra et al 2015 | United States | Urine | CLSI | | 103 | | 18 | | | 103 | | | 84 |
| 9 | Damiano et al 2021 | Tanzania | **N/P** | CLSI | | 15 | | 14 | | | 20 | | | 17 |
| 10 | Diriba et al 2020 | Ethiopia | Stool and healthy food handlers | EUCAST | | 119 | | 29 | | | 27 | | | 8 |
| 11 | Du et al 2002 | China | Blood | CLSI | | 60 | | 16 | | | 25 | | | 7 |
| 12 | Fatima et al 2018 | Pakistan | Urine, Pus, stool, Sputum, Throat swab, HVS, Ascitic Fluid, Cystic fluid, semen | CLSI and double disc synergy test | | 352 | | 79 | | | 352 | | | 16 |
| 13 | Han et al 2014 | Republic of Korea | Blood | VITEK2 automated system | | 20 | | 9 | | 20 | | | 12 | |
| 14 | Han et al 2015 | Republic of Korea | Urine | VITEK2 automated system | | 204 | | 21 | | 7 | | | 1 | |
| 15 | Harris et al 2015 | Singapore | Blood | EUCAST | | 476 | | 79 | | | 476 | | | 13 |
| 16 | Hyle et al 2004 | Pennsylvania | **N/P** | NCCLS | | 361 | | 151 | | | 361 | | | 183 |
| 17 | Jiménez-Guerra et al 2017 | Spain | Urine | CLSI and MicroScan system® | | 9772 | | 1027 | | | 1784 | | | 250 |
| 18 | Kader et al 2005 | Saudi Arabia | Urine, wounds, bed sores, blood, sputum, and others | MIC | | 1674 | | 173 | | | 781 | | | 95 |
| 19 | Kammili et al 2014 | India | rectal/faecal | disc diffusion | | 125 | | 23 | | | 89 | | | 30 |
| 20 | Kang et al 2004 | Korea | Blood | NCCLS and MIC | | 982 | | 76 | | | 471 | | | 75 |
| 21 | Karki et al 2021 | Nepal | Urine, blood, pus, sputum, body fluids, wound swab, tissue, appendicular, sites, catheter, stool, urethral, vaginal swabs,bone, and bone marrow aspirate | CLSI | | 455 | | 302 | | | 455 | | | 41 |
| 22 | Kayastha et al 2020 | Nepal | Urine, blood and pus | CLSI | | 79 | | 22 | | | 18 | | | 6 |
| 23 | Kernéis et al 2015 | France | Urine, and blood | MIC | | 33 | | 19 | | | 33 | | | 14 |
| 24 | Kim et al 2014 | Korea | Ascites and blood | CLSI [VITEK2] | | 182 | | 47 | | | 49 | | | 5 |
| 25 | Lee 2007 | Korea | Blood, pleural fluid and cerebrospinal fluid | disc diffusion test, MICs [Etest] CLSI | | 113 | | 28 | | | 119 | | | 54 |
| 26 | Lee wet al 2012 | Taiwan | Blood | CLSI | | 251 | | 111 | | | 251 | | | 140 |
| 27 | Lemarie et al 2009 | Thailand | Urine and blood | **N/P** | | 1959 | | 657 | | | 1185 | | | 676 |
| 28 | Letara et al 2021 | Tanzania | Rectal swab and urine | double disc synergy | | 278 | | 76 | | | 17 | | | 14 |
| 29 | Liang et al 2021 | China | Blood | VITEK 2 | | 301 | | 159 | | | 149 | | | 45 |
| 30 | Liu et al 2011 | Taiwan | Urine | CLSI MIC | | 200 | | 134 | | | 200 | | | 66 |
| 31 | Lowe et al 2012 | Canada | Urine and blood | CLSI | | 1994 | | 1,736 | | | 1994 | | | 258 |
| 32 | Mendelson et al 2005 | Israel | Urine | 350 | 77 | | 84 | | 34 | | |  |  |  |
| 33 | Ozgunes et al 2006 | Turkey | Various specimen | double-disk synergy | | 100 | | 12 | | | 100 | | | 47 |
| 34 | Quirante et al 2011 | Spain | Blood | Vitek system, CLSI | | 4,172 | | 1218 | | | 4,172 | | | 226 |
| 35 | Rahman et al 2004 | Bangladesh | Urine, pus, sputum, blood, vaginal swab and throat swab | NCCLS | | 81 | | 35 | | | 76 | | | 30 |
| 36 | Samaha-Kfoury et al 2005 | Lebanon | **N/P** | E-test | | 2000 | | 84 | | | 558 | | | 58 |
| 37 | Sawatwong et al 2019 | Thailand | Blood | disk diffusion | | 3,293 | | 883 | | | 1,059 | | | 290 |
| 38 | Shaikh et al 2014 | Indian | Urine, blood, sputum, eye discharges, surface swabs and body fluids | MIC | | 346 | | 93 | | | 346 | | | 74 |
| 39 | Shaikh et al 2010 | India | nose, pharynx and rectum | MIC | | 469 | | 97 | | | 203 | | | 58 |
| 40 | Shanthi et al 2010 | India | Urine, blood, exudates and respiratory secretions | CLSI | | 101 | | 49 | | | 101 | | | 19 |
| 41 | Sharif et al 2016 | Iran | Urine, stool, blood, peritoneal, and pleural fluids | Epsilometer test (E-test) and CLSI | | 134 | | 54 | | | 116 | | | 48 |
| 42 | Superti et al 2009 | Brazil | Blood | CLSI | | 81 | | 6 | | | 64 | | | 45 |
| 43 | Taneja et al 2010 | India | Blood | E-test | | 24 | | 10 | | | 64 | | | 41 |
| 44 | Tola et al 2021 | Ethiopia | Fecal/rectal swab | VITEK 2 ESBL | | 47 | | 39 | | | 47 | | | 8 |
| 45 | Tulara, 2018 | India | Urine | CLSI | | 384 | | 145 | | | 80 | | | 53 |
| 46 | Vargas et a 2009 | Brazil | Blood | CLSI | | 64 | | 6 | | | 81 | | | 55 |
| 47 | Viet et al 2021 | Vietnam | Vaginal swab | CLSI | | 432 | | 340 | | | 432 | | | 88 |
| 48 | Wang et al 2011 | Taiwan | **N/P** | CLSI | | 113 | | 67 | | | 113 | | | 45 |
| 49 | Wang et al 2019 | China | Blood, urine, wound, abscess, respiratory tract and skin | CLSI | | 498 | | 188 | | | 703 | | | 113 |
| 50 | Yang et al 2010 | Taiwan | Urine | CLSI | | 58 | | 35 | | | 58 | | | 23 |
| 51 | Yilmaz et al 2008 | Turkey | Urine | CLSI and Phoenix System | | 106 | | 52 | | | 16 | | | 10 |
| 52 | Yousef et al 2016 | Saudi Arabia | Urine | CLSI [MIC] | | 520 | | 218 | | | 160 | | | 78 |
| 53 | Dissanayake et al 2012 | Sri Lanka | Urine | CLSI | | 228 | | 66 | | | 58 | | | 29 |
| 54 | Leistner et al 2014 | Germany | Blood | Vitek 2 MIC | | 1,499 | | 66 | | | 352 | | | 178 |
| 55 | Abrar et al 2017 | Pakistan |  | Etest MIC | | 638 | | 294 | | | 338 | | | 108 |
| 56 | Alfaresi et al 2011 | United Arab Emirates (UAE) | Urine specimens, from blood, tracheal/bronchial aspirates, wound swabs, sputum, and ear discharges | PCR assays and CLSI | | 662 | | 150 | | | 662 | | | 90 |
| 57 | Barguigua et al 2011 | Morocco | Urinary tract infections | PCR assays and DDST | | 767 | | 10 | | | 36 | | | 2 |
| 58 | Baroud et al 2013 | Lebanon | Not stated | PCR assays, Etest MIC | | 2243 | | 24 | | | 572 | | | 14 |
| 59 | Doi et al 2012 | USA | Rectal swabs | PCR assays and Etest MIC | | 12 | | 7 | | | 37 | | | 23 |
| 60 | Edelstein et al 2003 | Russian | **N/P** | PCR assays, and NCCLS | | 494 | | 78 | | | 410 | | | 248 |
| 61 | Kim et al 2002 | Korea | **N/P** | PCR assays, ERIC-PCR | | 89 | | 15 | | | 68 | | | 34 |
| 62 | Kiratisin et al 2008 | Thailand | **N/P** | PCR assays, NCCLS MIC | | 362 | | 235 | | | 362 | | | 127 |
| 63 | Kizilates et al 2021 | Turkey | Rectal swab | real-time multiplex PCR and Etest MIC | | 67 | | 39 | | | 67 | | | 28 |
| 64 | Krishnamurthy et al 2013 | India | **N/P** | PCR assays and CLSI | | 57 | | 38 | | | 87 | | | 28 |
| 65 | Lee et al 2007 | Korea | **N/P** | PCR assays, Etest MIC | | 113 | | 28 | | | 119 | | | 54 |
| 66 | Nakama et al 2016 | Japan | Feces, sputum, urine, and vaginal discharge | PCR assays | | 121 | | 90 | | | 121 | | | 19 |
| 67 | Olalekan et al 2019 | Nigeria | Urine, wound, blood culture, lower respiratory tract and others | isothermal amplification, PCR and WGS | | 194 | | 113 | | | 193 | | | 62 |
| 68 | Pornsinchai et al 2015 | Thailand | Rectal swab | 16 | | 78 | | 74 | | | 78 | | | 4 |
| 69 | Priyadharshana et al 2019 | Sri Lanka | Urine | multiplex PCR, CLSI | | 149 | | 68 | | | 16 | | | 4 |
| 70 | Seputiene et al 2010 | Lithuania | Bronchial, urine, surgical and blood |  | | 175 | | 62 | | | 175 | | | 113 |
| 71 | Siu et al 1999 | Taiwan | **N/P** | PCR assays, NCCLS MIC | | 113 | | 21 | | | 87 | | | 49 |
| 72 | Thirapanmethee et al 2014 | Thailand | Ear–nose–throat, intensive care unit, outpatient department, pediatric intensive care unit, surgical and intensive care unit. | DDST, PCR and LAMP | | 35 | | 27 | | | 35 | | | 8 |
| 73 | Tofteland et al 2007 | Norway | **N/P** |  | | 89 | | 50 | | | 27 | | | 19 |
| 74 | Zerr et al 2016 | USA | Urine | CLSI and PCR | | 1058 | | 178 | | | 146 | | | 32 |
| 75 | ABE et al 2021 | Côte d'Ivoire | Urine | PCR and double synergistic | | 30 | | 17 | | | 9 | | | 4 |
| 76 | Amer et al 2019 | Egypt | Urine, pus, blood, sputum, semen and stool | DDST and combination disk test (CDT) | | 83 | | 61 | | | 85 | | | 47 |
| 77 | Chirindzeet al 2018 | Mozambique | Stool | CLSI, double disc synergy | | 55 | | 34 | | | 55 | | | 21 |
| 78 | Deji-Agboolaet al 2020 | Nigeria | Stool and urine | DDS20 | | 256 | | 19 | | | 89 | | | 11 |
| 79 | Ghenea et al 2022 | Romania | Purulent secretions, catheters, peritoneal fluids, tracheal aspirates, and sputum | CLSI, VITEK2 system | | 46 | | 14 | | | 46 | | | 34 |
| 80 | Hansen et al 2012 | Denmark | Blood and urine | Vitek 2 | | 12382 | | 205 | | | 1751 | | | 73 |
| 81 | Hasani et al 2017 | Iran | Blood, wounds, urine, endotracheal tubes, and various bodily fluids | CLSI | | 71 | | 41 | | | 63 | | | 45 |
| 82 | Jahromy et al 2019 | Iran | Urine | CLSI, MIC | | 237 | | 138 | | | 47 | | | 34 |
| 83 | Juma et al 2016 | Kenya | Stool | CLSI, E-test | | 100 | | 15 | | | 200 | | | 3 |
| 84 | Karim et al 2017 | Bangladesh | Blood, swab and urine | CLSI | | 85 | | 36 | | | 85 | | | 31 |
| 85 | Khanal et al 2022 | Nepal | Urine | CLSI | | 360 | | 58 | | | 42 | | | 2 |
| 86 | Kim et al 2005 | Korea | **N/P** | CLSI, MIC | | 272 | | 32 | | | 158 | | | 28 |
| 87 | Mulvey et al 2004 | Canada | Blood, wound and abscess | NCCLS | | 425 | | 74 | | | 80 | | | 37 |
| 88 | Ogbolu et al 2013 | Nigeria | Faecal | CLSI, MIC | | 54 | | 12 | | | 60 | | | 6 |
| 89 | Riaz et al 2015 | Pakistan | Blood, wound swabs | CLSI, MIC | | 662 | | 118 | | | 153 | | | 78 |
| 90 | Somily et al 2015 | Saudi Arabia | Blood, wound swabs, endotracheal secretions, sputum, body fluids, and urine | CLSI, E-test | | 77 | | 50 | | | 77 | | | 27 |
| 91 | Ushie et al 2020 | Nigeria | Urine | DDST | | 10 | | 9 | | | 16 | | | 14 |
| 92 | Yarima et al 2020 | Nigeria | Urine | DDST | | 100 | | 40 | | | 109 | | | 59 |
| 93 | Adeyankinnu et al 2014 | Nigeria | Urine, wound swabs, aspirates, blood, high vaginal and endocervical swabs, and sputum | CLSI | | 197 | | 135 | | | 197 | | | 62 |
| 94 | Ambrose et al 2003 | United States and Canada | Various specimen | NCCLS, Etest strips | | 1909 | | 65 | | | 743 | | | 40 |
| 95 | Arbab et al 2012 | Iran | Urine | CLSI, Double disk | | 885 | | 110 | | | 110 | | | 33 |
| 96 | Aziz et al 2019 | Pakistan | Urine | (NCCLS | | 163 | | 96 | | | 163 | | | 67 |
| 97 | Mahomed and Coovadia, 2015. | South Africa | Stools and urine | CLSI, VITEK 2, | | 97 | | 3 | | | 12 | | | 11 |
| 98 | Mofolorunsho et al 2021 | Nigeria | Urine | CLSI | | 78 | | 54 | | | 78 | | | 24 |
| 99 | Ngoi et al 2021 | Malaysia | **N/P** | CLSI | | 50 | | 28 | | | 50 | | | 25 |
| 100 | Onanuga et al 2019 | Nigeria | Urine | CLSI | | 64 | | 37 | | | 108 | | | 44 |
| 101 | Siriphap et al 2022 | Thailand | Urine. sputum, blood, pus and body fluids | CLSI | | 11,065 | | 4,706 | | | 5,617 | | | 1,697 |

**Table S2.** List and characteristics of eligible studies included in the meta-analysis with respect to ESBL-producing *Escherichia coli* and *Klebsiella pneumoniae* in animals.

|  | **Authors & years** | **Country** | **Samples type** | **ESBL diagnostic Methods** | ***E. coli*** | | ***K. pneumoniae*** | |
| --- | --- | --- | --- | --- | --- | --- | --- | --- |
|  |  |  |  |  | **Isolates** | **Prevalence of ESBL** | **Isolates** | **Prevalence of ESBL** |
| 1 | Badri et al 2017 | Sudan | Milk | DDST | 22 | 16 | 36 | 24 |
| 1 | Bandyopadhyay et al 2021 | India | rectal swabs | CLSI | 61 | 35 | 61 | 37 |
| 3 | Chenouf et al 2020 | Algeria | Broiler livers | CLSI, MIC | 73 | 8 | 5 | 5 |
| 4 | Chijioke et al 2022 | Nigeria | Retail meat viz: beef, chicken, and goat meat | DDST | 108 | 20 | 64 | 9 |
| 5 | Eibach et al 2018 | Ghana | Poultry meat | VITEK 2 | 70 | 46 | 70 | 35 |
| 6 | Gundogan and Avci 2013 | Turkey | Foods of animal origin | CLSI | 45 | 20 | 13 | 5 |
| 7 | Hiroi et al 2011 | Japan | Food-Producing Animals | CLSI | 109 | 48 | 1 | 1 |
| 8 | Jamborova et al 2018 | Canada | Birds | CLSI | 449 | 96 | 449 | 3 |
| 9 | Johansson et al 2022 | Finland | Dogs | MIC | 60 | 58 | 60 | 2 |
| 10 | Kuan et al 2016 | Taiwan | URINE OF DOGS AND CATS | CLSI | 60 | 2 | 22 | 5 |
| 11 | Mbah et al 2021 | Nigeria | Swab sticks chickens | DDST | 156 | 135 | 156 | 14 |
| 12 | Montso et al 2019 | South Africa | Raw Beef | CLSI | 145 | 69 | 114 | 35 |
| 13 | Mwanginde et al 2021 | Tanzania | Poultry meat vendors | DDST | 101 | 84 | 29 | 23 |
| 14 | Sai’du et al 2022 | Nigeria | Chicken Meat | Disk diffusion method | 178 | 14 | 28 | 5 |
| 15 | Saidani et al 2019 | Tunisia | Camels | MIC | 232 | 163 | 232 | 16 |
| 16 | Sivaraman et al 2020 | India | Fish | CLSI | 66 | 54 | 66 | 12 |
| 17 | Vo et al 2007 | Netherland | Horses | CLSI | 1347 | 4 | 1347 | 3 |

**Table S3.** List and characteristics of eligible studies included in the meta-analysis with respect to ESBL-producing *Escherichia coli* and *Klebsiella pneumoniae* in environment.

|  | **Authors & years** | **Country** | **Samples type** | **ESBL diagnostic Methods** | ***E. coli*** | | ***K. pneumoniae*** | |
| --- | --- | --- | --- | --- | --- | --- | --- | --- |
|  |  |  |  |  | **Isolates** | **Prevalence of ESBL** | **Isolates** | **Prevalence of ESBL** |
| 1 | Atta et al 2022 | Nigeria | Water | CLSI | 3 | 13 | 3 | 9 |
| 2 | Devi et al 2020 | India | Manure | CLSI | 491 | 1080 | 85 | 1080 |
| 3 | Hassen et al 2020 | Tunisia | Water | CLSI | 33 | 37 | 4 | 37 |
| 4 | Mahato et al 2019 | Nepal | Hospitals sewage | CLSI | 6 | 7 | 2 | 6 |
| 5 | Romyasamit et al 2021 | Thailand | Raw vegetables | CLSI | 5 | 14 | 9 | 14 |

**Table S4.** List and characteristics of eligible studies included in the meta-analysis with respect to ESBL-producing *Escherichia coli* and *Klebsiella pneumoniae* from the animals and the environment.

|  | **Authors & years** | **Country** | **Samples type** | **ESBL diagnostic Methods** | ***E. coli*** | | ***K. pneumoniae*** | |
| --- | --- | --- | --- | --- | --- | --- | --- | --- |
|  |  |  |  |  | **Isolates** | **Prevalence of ESBL** | **Isolates** | **Prevalence of ESBL** |
| 1 | Sivaraman et al 2021 | India | shrimp and water | CLSI | 261 | 32 | 261 | 15 |
| 2 | Soré et al 2021 | Burkina Faso | cloacal swabs, water, food, farms space | DDST | 51 | 43 | 51 | 13 |
| 3 | Kurittu et al 2021 | Finland | vegetables, fruits and berries, meat, and seafood products | CLSI | 313 | 21 | 313 | 5 |

**Reference**

1. Bayraktar, B., Pelit, S., Bulut, M.E. and Aktaş, E., 2019. Trend in antibiotic resistance of extended-spectrum beta-lactamase-producing Escherichia coli and Klebsiella pneumoniae bloodstream infections. *The Medical Bulletin of Sisli Etfal Hospital*, *53*(1), p.70.
2. Bazzaz, B., Naderinasab, M., Mohamadpoor, A., Farshadzadeh, Z., Ahmadi, S. and Yousefi, F., 2009. The prevalence of extended-spectrum beta-lactamase-producing Escherichia coli and Klebsiella pneumoniae among clinical isolates from a general hospital in Iran. *Acta microbiologica et immunologica Hungarica*, *56*(1), pp.89-99.
3. Ben-Chetrit, E., Eldaim, M.A., Bar-Meir, M., Dodin, M. and Katz, D.E., 2019. Associated factors and clinical outcomes of bloodstream infection due to extended-spectrum β-lactamase-producing Escherichia coli and Klebsiella pneumoniae during febrile neutropenia. *International journal of antimicrobial agents*, *53*(4), pp.423-428.
4. Bishara, J., Livne, G., Ashkenazi, S., Levy, I., Pitlik, S., Ofir, O., Lev, B. and Samra, Z., 2005. Antibacterial susceptibility of extended-spectrum beta-lactamase-producing Klebsiella pneumoniae and Escherichia coli. *Isr Med Assoc J*, *7*(5), pp.298-301.
5. Chander, A. and Shrestha, C.D., 2013. Prevalence of extended spectrum beta lactamase producing Escherichia coli and Klebsiella pneumoniae urinary isolates in a tertiary care hospital in Kathmandu, Nepal. *BMC Research notes*, *6*(1), pp.1-6.
6. Cheng, W.L., Hsueh, P.R., Lee, C.C., Li, C.W., Li, M.J., Chang, C.M., Lee, N.Y. and Ko, W.C., 2016. Bacteremic pneumonia caused by extended-spectrum beta-lactamase-producing Escherichia coli and Klebsiella pneumoniae: appropriateness of empirical treatment matters. *Journal of Microbiology, Immunology and Infection*, *49*(2), pp.208-215.
7. Cho, Y.H., Jung, S.I., Chung, H.S., Yu, H.S., Hwang, E.C., Kim, S.O., Kang, T.W., Kwon, D.D. and Park, K., 2015. Antimicrobial susceptibilities of extended-spectrum beta-lactamase-producing Escherichia coli and Klebsiella pneumoniae in health care-associated urinary tract infection: focus on susceptibility to fosfomycin. *International urology and nephrology*, *47*, pp.1059-1066.
8. Chopra, T., Marchaim, D., Johnson, P.C., Chalana, I.K., Tamam, Z., Mohammed, M., Alkatib, S., Tansek, R., Chaudhry, K., Zhao, J.J. and Pogue, J.M., 2015. Risk factors for bloodstream infection caused by extended-spectrum β-lactamase–producing Escherichia coli and Klebsiella pneumoniae: a focus on antimicrobials including cefepime. *American journal of infection control*, *43*(7), pp.719-723.
9. Damiano, P., Salema, E.J. and Silago, V., 2021. The susceptibility of multidrug resistant and biofilm forming Klebsiella pneumoniae and Escherichia coli to antiseptic agents used for preoperative skin preparations at zonal referral hospital in Mwanza, Tanzania. *Malawi Medical Journal*, *33*(1), pp.59-64.
10. Diriba, K., Awulachew, E., Tekele, L. and Ashuro, Z., 2020. Fecal carriage rate of extended-spectrum beta-lactamase-producing Escherichia coli and Klebsiella pneumoniae among apparently health food handlers in Dilla University student cafeteria. *Infection and Drug Resistance*, pp.3791-3800.
11. Du, B., Long, Y., Liu, H., Chen, D., Liu, D., Xu, Y. and Xie, X., 2002. Extended-spectrum beta-lactamase-producing Escherichia coli and Klebsiella pneumoniae bloodstream infection: risk factors and clinical outcome. *Intensive care medicine*, *28*, pp.1718-1723.
12. Fatima, S., Muhammad, I.N., Khan, M.N. and Jamil, S., 2018. Phenotypic expression and prevalence of multi drug resistant extended spectrum beta-lactamase producing Escherichia coli and Klebsiella pneumoniae in Karachi, Pakistan. *Pak J Pharm Sci*, *31*(4), pp.1379-84.
13. Harris, P.N., Yin, M., Jureen, R., Chew, J., Ali, J., Paynter, S., Paterson, D.L. and Tambyah, P.A., 2015. Comparable outcomes for β-lactam/β-lactamase inhibitor combinations and carbapenems in definitive treatment of bloodstream infections caused by cefotaxime-resistant Escherichia coli or Klebsiella pneumoniae. *Antimicrobial resistance and infection control*, *4*, pp.1-10.
14. Han, S.B., Lee, S.C., Lee, S.Y., Jeong, D.C. and Kang, J.H., 2015. Aminoglycoside therapy for childhood urinary tract infection due to extended-spectrum β-lactamase-producing Escherichia coli or Klebsiella pneumoniae. *BMC infectious diseases*, *15*(1), pp.1-8.
15. Hyle, E.P., Lipworth, A.D., Zaoutis, T.E., Nachamkin, I., Fishman, N.O., Bilker, W.B., Mao, X. and Lautenbach, E., 2005. Risk factors for increasing multidrug resistance among extended-spectrum β-lactamase-producing Escherichia coli and Klebsiella species. *Clinical infectious diseases*, *40*(9), pp.1317-1324.
16. Kader, A.A. and Kumar, A., 2005. Prevalence and antimicrobial susceptibility of extended-spectrum β-lactamase-producing Escherichia coli and Klebsiella pneumoniae in a general hospital. *Annals of Saudi medicine*, *25*(3), pp.239-242.
17. Kammili, N., Cherukuri, N., Palvai, S., Pazhni, G., Ramamurthy, T., Rao, J. and Anuradha, P., 2014. Molecular epidemiology of extended spectrum [beta]-lactamase-producing Escherichia coli and Klebsiella pneumoniae in a tertiary care hospital. *Indian Journal of Medical Microbiology*, *32*(2), p.205.
18. Kang, C.I., Kim, S.H., Park, W.B., Lee, K.D., Kim, H.B., Oh, M.D., Kim, E.C., Lee, H.S. and Choe, K.W., 2004. Clinical outcome of bacteremic spontaneous bacterial peritonitis due to extended-spectrum beta-lactamase-producing Escherichia coli and Klebsiella pneumoniae. *The Korean journal of internal medicine*, *19*(3), p.160.
19. Karki, D., Dhungel, B., Bhandari, S., Kunwar, A., Joshi, P.R., Shrestha, B., Rijal, K.R., Ghimire, P. and Banjara, M.R., 2021. Antibiotic resistance and detection of plasmid mediated colistin resistance mcr-1 gene among Escherichia coli and Klebsiella pneumoniae isolated from clinical samples. *Gut pathogens*, *13*(1), pp.1-16.
20. Kayastha, K., Dhungel, B., Karki, S., Adhikari, B., Banjara, M.R., Rijal, K.R. and Ghimire, P., 2020. Extended-spectrum β-lactamase-producing Escherichia coli and Klebsiella species in pediatric patients visiting International Friendship Children’s Hospital, Kathmandu, Nepal. *Infectious Diseases: Research and Treatment*, *13*, p.1178633720909798.
21. Kim, M.J., Song, K.H., Kim, N.H., Choe, P.G., Park, W.B., Bang, J.H., Kim, E.S., Park, S.W., Kim, H.B., Lee, H.S. and Oh, M.D., 2014. Clinical outcomes of spontaneous bacterial peritonitis due to extended-spectrum beta-lactamase-producing Escherichia coli or Klebsiella pneumoniae: a retrospective cohort study. *Hepatology international*, *8*, pp.582-587.
22. Lee, J., Pai, H., Kim, Y.K., Kim, N.H., Eun, B.W., Kang, H.J., Park, K.H., Choi, E.H., Shin, H.Y., Kim, E.C. and Lee, H.J., 2007. Control of extended-spectrum β-lactamase-producing Escherichia coli and Klebsiella pneumoniae in a children's hospital by changing antimicrobial agent usage policy. *Journal of antimicrobial Chemotherapy*, *60*(3), pp.629-637.
23. Letara, N., Ngocho, J.S., Karami, N., Msuya, S.E., Nyombi, B., Kassam, N.A., Skovbjerg, S., Åhren, C., Philemon, R. and Mmbaga, B.T., 2021. Prevalence and patient related factors associated with Extended-Spectrum Beta-Lactamase producing Escherichia coli and Klebsiella pneumoniae carriage and infection among pediatric patients in Tanzania. *Scientific Reports*, *11*(1), p.22759.
24. Liang, T., Xu, C., Cheng, Q., Tang, Y., Zeng, H. and Li, X., 2021. Epidemiology, risk factors, and clinical outcomes of bloodstream infection due to extended-spectrum beta-lactamase-producing Escherichia coli and Klebsiella pneumoniae in hematologic malignancy: a retrospective study from Central South China. *Microbial Drug Resistance*, *27*(6), pp.800-808.
25. Lin, J.N., Chen, Y.H., Chang, L.L., Lai, C.H., Lin, H.L. and Lin, H.H., 2011. Clinical characteristics and outcomes of patients with extended-spectrum β-lactamase-producing bacteremias in the emergency department. *Internal and emergency medicine*, *6*, pp.547-555.
26. Liu, H.Y., Lin, H.C., Lin, Y.C., Yu, S.H., Wu, W.H. and Lee, Y.J., 2011. Antimicrobial susceptibilities of urinary extended-spectrum beta-lactamase-producing Escherichia coli and Klebsiella pneumoniae to fosfomycin and nitrofurantoin in a teaching hospital in Taiwan. *Journal of Microbiology, Immunology and Infection*, *44*(5), pp.364-368.
27. Lowe, C.F., McGeer, A., Muller, M.P. and Katz, K., 2012. Decreased susceptibility to noncarbapenem antimicrobials in extended-spectrum-β-lactamase-producing Escherichia coli and Klebsiella pneumoniae isolates in Toronto, Canada. *Antimicrobial agents and chemotherapy*, *56*(7), pp.3977-3980.
28. Mendelson, G., Hait, V., Ben-Israel, J., Gronich, D., Granot, E. and Raz, R., 2005. Prevalence and risk factors of extended-spectrum beta-lactamase-producing Escherichia coli and Klebsiella pneumoniae in an Israeli long-term care facility. *European journal of clinical microbiology and infectious diseases*, *24*, pp.17-22.
29. Ozgunes, I., Erben, N., Kiremitci, A., Kartal, E.D., Durmaz, G., Colak, H., Usluer, G. and Colak, E., 2006. The prevalence of extended-spectrum beta lactamase-producing Escherichia coli and Klebsiella pneumoniae in clinical isolates and risk factors. *Saudi medical journal*, *27*(5), p.608.
30. Quirante, O.F., Cerrato, S.G. and Pardos, S.L., 2011. Risk factors for bloodstream infections caused by extended-spectrum β-lactamase-producing Escherichia coli and Klebsiella pneumoniae. *The Brazilian Journal of Infectious Diseases*, *15*(4), pp.370-376
31. Rahman, M.M., Haq, J.A., Hossain, M.A., Sultana, R., Islam, F. and Islam, A.S., 2004. Prevalence of extended-spectrum β-lactamase-producing Escherichia coli and Klebsiella pneumoniae in an urban hospital in Dhaka, Bangladesh. *International journal of antimicrobial agents*, *24*(5), pp.508-510.
32. Sawatwong, P., Sapchookul, P., Whistler, T., Gregory, C.J., Sangwichian, O., Makprasert, S., Jorakate, P., Srisaengchai, P., Thamthitiwat, S., Promkong, C. and Nanvatthanachod, P., 2019. High burden of extended-spectrum β-lactamase–producing Escherichia coli and klebsiella pneumoniae bacteremia in older adults: A seven-year study in two rural Thai provinces. *The American journal of tropical medicine and hygiene*, *100*(4), p.943.
33. Shakil, S., Akram, M., Ali, S.M. and Khan, A.U., 2010. Acquisition of extended-spectrum β-lactamase producing Escherichia coli strains in male and female infants admitted to a neonatal intensive care unit: molecular epidemiology and analysis of risk factors. *Journal of medical microbiology*, *59*(8), pp.948-954.
34. Shanthi, M. and Sekar, U., 2010. Extended spectrum beta lactamase producing Escherichia coli and Klebsiella pneumoniae: risk factors for infection and impact of resistance on outcomes. *J Assoc Physicians India*, *58*(Suppl), pp.41-44.
35. Superti, S.V., Augusti, G. and Zavascki, A.P., 2009. Risk factors for and mortality of extended-spectrum-β-lactamase-producing Klebsiella pneumoniae and Escherichia coli nosocomial bloodstream infections. *Revista do Instituto de Medicina Tropical de São Paulo*, *51*, pp.211-216.
36. Taneja, J., Mishra, B., Thakur, A., Dogra, V. and Loomba, P., 2010. Nosocomial blood-stream infections from extended-spectrum-beta-lactamase-producing Escherichia coli and Klebsiella pneumonia from GB Pant Hospital, New Delhi. *The Journal of Infection in Developing Countries*, *4*(08), pp.517-520.
37. Tola, M.A., Abera, N.A., Gebeyehu, Y.M., Dinku, S.F. and Tullu, K.D., 2021. High prevalence of extended-spectrum beta-lactamase-producing Escherichia coli and Klebsiella pneumoniae fecal carriage among children under five years in Addis Ababa, Ethiopia. *PloS one*, *16*(10), p.e0258117.
38. Tulara, N.K., 2018. Nitrofurantoin and fosfomycin for extended spectrum beta-lactamases producing Escherichia coli and Klebsiella pneumoniae. *Journal of global infectious diseases*, *10*(1), p.19.
39. Vargas Superti, S., Augusti, G. and Prehn ZavasckI, A., 2009. Risk factors for and mortality of extended-spectrum-β-lactamase-producing Klebsiella pneumoniae and Escherichia coli nosocomial bloodstream infections. *Revista do Instituto de Medicina Tropical de São Paulo*, *51*(4), pp.211-216.
40. Viet, N.T., Van Du, V., Thuan, N.D., Van Tong, H., Toan, N.L., Van Mao, C., Van Tuan, N., Pallerla, S.R., Nurjadi, D., Velavan, T.P. and Son, H.A., 2021. Maternal vaginal colonization and extended-spectrum beta-lactamase-producing bacteria in Vietnamese pregnant women. *Antibiotics*, *10*(5), p.572.
41. Wang, S.S., Lee, N.Y., Hsueh, P.R., Huang, W.H., Tsui, K.C., Lee, H.C., Wu, C.J., Chang, C.M., Huang, C.C., Huang, C.F. and Ko, W.C., 2011. Clinical manifestations and prognostic factors in cancer patients with bacteremia due to extended-spectrum β-lactamase-producing Escherichia coli or Klebsiella pneumoniae. *Journal of Microbiology, Immunology and Infection*, *44*(4), pp.282-288.
42. Wang, Y., Zhang, Q., Jin, Y., Jin, X., Yu, J. and Wang, K., 2019. Epidemiology and antimicrobial susceptibility profiles of extended-spectrum beta-lactamase–producing Klebsiella pneumoniae and Escherichiacoli in China. *Brazilian Journal of Microbiology*, *50*, pp.669-675.
43. Yang, Y.S., Ku, C.H., Lin, J.C., Shang, S.T., Chiu, C.H., Yeh, K.M., Lin, C.C. and Chang, F.Y., 2010. Impact of extended-spectrum β-lactamase-producing Escherichia coli and Klebsiella pneumoniae on the outcome of community-onset bacteremic urinary tract infections. *Journal of Microbiology, Immunology and Infection*, *43*(3), pp.194-199.
44. Yilmaz, E., Akalin, H., Özbey, S., Kordan, Y., Sinirtaş, M., Gürcüoglu, E., Özakin, C., Heper, Y., Mistik, R. and Helvaci, S., 2008. Risk factors in community-acquired/onset urinary tract infections due to extended-spectrum beta-lactamase-producing Escherichia coli and Klebsiella pneumoniae. *Journal of Chemotherapy*, *20*(5), pp.581-585.
45. Abrar, S., Vajeeha, A., Ul-Ain, N. and Riaz, S., 2017. Distribution of CTX-M group I and group III β-lactamases produced by Escherichia coli and klebsiella pneumoniae in Lahore, Pakistan. *Microbial pathogenesis*, *103*, pp.8-12.
46. Alfaresi, M.S., Elkoush, A.A., Alshehhi, H.M. and Abdulsalam, A.I., 2011. Molecular characterization and epidemiology of extended-spectrum beta-lactamase-pro ducing Escherichia coli and Klebsiella pneumoniae isolates in the United Arab Emirates. *Medical Principles and Practice*, *20*(2), pp.177-180.
47. Barguigua, A., El Otmani, F., Talmi, M., Bourjilat, F., Haouzane, F., Zerouali, K. and Timinouni, M., 2011. Characterization of extended-spectrum β-lactamase-producing Escherichia coli and Klebsiella pneumoniae isolate s from the community in Morocco. *Journal of medical microbiology*, *60*(9), pp.1344-1352.
48. Baroud, Á., Dandache, I., Araj, G.F., Wakim, R., Kanj, S., Kanafani, Z., Khairallah, M., Sabra, A., Shehab, M., Dbaibo, G. and Matar, G.M., 2013. Underlying mechanisms of carbapenem resistance in extended-spectrum β-lactamase-producing Klebsiella pneumoniae and Escherichia coli isolates at a tertiary care centre in Lebanon: role of OXA-48 and NDM-1 carbapenemases. *International journal of antimicrobial agents*, *41*(1), pp.75-79.
49. Doi, Y., Adams-Haduch, J.M., Peleg, A.Y. and D'Agata, E.M., 2012. The role of horizontal gene transfer in the dissemination of extended-spectrum beta-lactamase–producing Escherichia coli and Klebsiella pneumoniae isolates in an endemic setting. *Diagnostic microbiology and infectious disease*, *74*(1), pp.34-38.
50. Edelstein, M., Pimkin, M., Palagin, I., Edelstein, I. and Stratchounski, L., 2003. Prevalence and molecular epidemiology of CTX-M extended-spectrum β-lactamase-producing Escherichia coli and Klebsiella pneumoniae in Russian hospitals. *Antimicrobial agents and chemotherapy*, *47*(12), pp.3724-3732.
51. Kim, Y.K., Pai, H., Lee, H.J., Park, S.E., Choi, E.H., Kim, J., Kim, J.H. and Kim, E.C., 2002. Bloodstream infections by extended-spectrum β-lactamase-producing Escherichia coli and Klebsiella pneumoniae in children: epidemiology and clinical outcome. *Antimicrobial agents and chemotherapy*, *46*(5), pp.1481-1491.
52. Kiratisin, P., Apisarnthanarak, A., Laesripa, C. and Saifon, P., 2008. Molecular characterization and epidemiology of extended-spectrum-β-lactamase-producing Escherichia coli and Klebsiella pneumoniae isolates causing health care-associated infection in Thailand, where the CTX-M family is endemic. *Antimicrobial agents and chemotherapy*, *52*(8), pp.2818-2824.
53. Kizilates, F., Yakupogullari, Y., Berk, H., Oztoprak, N. and Otlu, B., 2021. Risk factors for fecal carriage of extended-spectrum beta-lactamase-producing and carbapenem-resistant Escherichia coli and Klebsiella pneumoniae strains among patients at hospital admission. *American Journal of Infection Control*, *49*(3), pp.333-339.
54. Krishnamurthy, V., Vijaykumar, G.S., Kumar, S., Prashanth, H.V., Prakash, R. and Nagaraj, E.R., 2013. Phenotypic and genotypic methods for detection of extended spectrum β lactamase producing Escherichia coli and Klebsiella pneumoniae isolated from ventilator associated pneumonia. *Journal of clinical and diagnostic research: JCDR*, *7*(9), p.1975.
55. Nakama, R., Shingaki, A., Miyazato, H., Higa, R., Nagamoto, C., Hamamoto, K., Ueda, S., Hachiman, T., Touma, Y., Miyagi, K. and Kawahara, R., 2016. Current status of extended spectrum β-lactamase-producing Escherichia coli, Klebsiella pneumoniae and Proteus mirabilis in Okinawa prefecture, Japan. *Journal of Infection and Chemotherapy*, *22*(5), pp.281-286.
56. Pornsinchai, P., Chongtrakool, P., Diraphat, P., Siripanichgon, K. and Malathum, K., 2015. emergency room: an unrecognized source of extended-spectrum [beta]-lactamase producing Escherichia coli and Klebsiella pneumoniae. *Southeast Asian Journal of Tropical Medicine and Public Health*, *46*(1), p.51.
57. Šeputienė, V., Linkevičius, M., Bogdaitė, A., Povilonis, J., Plančiūnienė, R., Giedraitienė, A., Pavilonis, A. and Sužiedėlienė, E., 2010. Molecular characterization of extended-spectrum β-lactamase-producing Escherichia coli and Klebsiella pneumoniae isolates from hospitals in Lithuania. *Journal of Medical Microbiology*, *59*(10), pp.1263-1265.
58. Siu, L.K., Lu, P.L., Hsueh, P.R., Lin, F.M., Chang, S.C., Luh, K.T., Ho, M. and Lee, C.Y., 1999. Bacteremia due to extended-spectrum β-lactamase-producing Escherichia coli and Klebsiella pneumoniae in a pediatric oncology ward: clinical features and identification of different plasmids carrying both SHV-5 and TEM-1 genes. *Journal of clinical microbiology*, *37*(12), pp.4020-4027
59. Priyadharshana, U., Piyasiri, L.B. and Wijesinghe, C., 2019. Prevalence, antibiotic sensitivity pattern and genetic analysis of extended spectrum beta lactamase producing Escherichia coli and Klebsiella spp among patients with community acquired urinary tract infection in Galle district, Sri Lanka. *Ceylon Medical Journal*, *64*(4), pp.140-1
60. Thirapanmethee, K., Pothisamutyothin, K., Nathisuwan, S., Chomnawang, M.T. and Wiwat, C., 2014. Loop‐mediated isothermal amplification assay targeting the blaCTX‐M9 gene for detection of extended spectrum β‐lactamase‐producing Escherichia coli and Klebsiella pneumoniae. *Microbiology and immunology*, *58*(12), pp.655-665.
61. Tofteland, S., Haldorsen, B., Dahl, K.H., Simonsen, G.S., Steinbakk, M., Walsh, T.R., Sundsfjord, A. and Norwegian ESBL Study Group, 2007. Effects of phenotype and genotype on methods for detection of extended-spectrum-β-lactamase-producing clinical isolates of Escherichia coli and Klebsiella pneumoniae in Norway. *Journal of clinical microbiology*, *45*(1), pp.199-205.
62. Zerr, D.M., Miles-Jay, A., Kronman, M.P., Zhou, C., Adler, A.L., Haaland, W., Weissman, S.J., Elward, A., Newland, J.G., Zaoutis, T. and Qin, X., 2016. Previous antibiotic exposure increases risk of infection with extended-spectrum-β-lactamase-and AmpC-producing Escherichia coli and Klebsiella pneumoniae in pediatric patients. *Antimicrobial agents and chemotherapy*, *60*(7), pp.4237-4243.
63. Dissanayake, D.M.B.T., Fernando, S.S.N. and Chandrasiri, N.S., 2012. The distribution and characteristics of Extended-Spectrum β-Lactamase producing Escherichia coli and Klebsiella species among urinary isolates in a tertiary care hospital. *Sri Lankan Journal of Infectious Diseases*, *2*(2).
64. Leistner, R., Gürntke, S., Sakellariou, C., Denkel, L.A., Bloch, A., Gastmeier, P. and Schwab, F., 2014. Bloodstream infection due to extended-spectrum beta-lactamase (ESBL)-positive K. pneumoniae and E. coli: an analysis of the disease burden in a large cohort. *Infection*, *42*, pp.991-997.
65. Ambrose, P.G., Bhavnani, S.M. and Jones, R.N., 2003. Pharmacokinetics-pharmacodynamics of cefepime and piperacillin-tazobactam against Escherichia coli and Klebsiella pneumoniae strains producing extended-spectrum β-lactamases: report from the ARREST program. *Antimicrobial Agents and Chemotherapy*, *47*(5), pp.1643-1646.
66. Arbabi, L., Rahbar, M., Jabbari, M., Mohammad-Zadeh, M., Azimi, L., Namvar, A.E. and Lari, A.R., 2012. Extended-spectrum β-lactamase-producing E. coli and Klebsiella pneumoniae isolated from urinary tract infections in Milad Hospital, Tehran, Iran. *HealthMED*, *6*, pp.2818-22.
67. Mahomed, S. and Coovadia, Y.M., 2015. Faecal carriage of Extended Spectrum Beta-lactamase producing Escherichia coli and Klebsiella Pneumoniae in children from the community of Kwadedangendlale, KwaZulu-Natal, South Africa. *International Journal of Infection Control*, *11*(3).
68. Mofolorunsho, K.C., Ocheni, H.O., Aminu, R.F., Omatola, C.A. and Olowonibi, O.O., 2021. Prevalence and antimicrobial susceptibility of extended-spectrum beta lactamases-producing Escherichia coli and Klebsiella pneumoniae isolated in selected hospitals of Anyigba, Nigeria. *African Health Sciences*, *21*(2), pp.505-512.
69. Ngoi, S.T., Teh, C.S.J., Chong, C.W., Abdul Jabar, K., Tan, S.C., Yu, L.H., Leong, K.C., Tee, L.H. and AbuBakar, S., 2021. In vitro efficacy of flomoxef against extended-spectrum beta-lactamase-producing Escherichia coli and Klebsiella pneumoniae associated with urinary tract infections in Malaysia. *Antibiotics*, *10*(2), p.181.
70. Onanuga, A., Vincent, C.H. and Eboh, D.D., 2019. Carbapenem Resistance among Extended Spectrum Beta-Lactamases Producing Escherichia coli and Klebsiella pneumoniae isolates from Patents with Urinary Tract Infections in Port-Harcourt, Nigeria. *Nigerian Journal of Pharmaceutical and Applied Science Research*, *8*(1), pp.16-23.
71. Siriphap, A., Kitti, T., Khuekankaew, A., Boonlao, C., Thephinlap, C., Thepmalee, C., Suwannasom, N. and Khoothiam, K., 2022. High prevalence of extended-spectrum beta-lactamase-producing Escherichia coli and Klebsiella pneumoniae isolates: A 5-year retrospective study at a Tertiary Hospital in Northern Thailand. *Frontiers in Cellular and Infection Microbiology*, p.1157.
72. Somily, A.M., Arshad, M.Z., Garaween, G.A. and Senok, A.C., 2015. Phenotypic and genotypic characterization of extended-spectrum β-lactamases producing Escherichia coli and Klebsiella pneumoniae in a tertiary care hospital in Riyadh, Saudi Arabia. *Annals of Saudi Medicine*, *35*(6), pp.435-439.
73. Riaz, S. and Bashir, M.F., 2015. Phenotypic and molecular characterization of plasmid-encoded extended spectrum beta-lactamases produced by Escherichia coli and Klebsiella spp from Lahore, Pakistan. *Tropical Journal of Pharmaceutical Research*, *14*(9), pp.1597-1604.
74. Ogbolu, D.O., Alli, O.T., Olanipekun, L.B., Ojo, O.I. and Makinde, O.O., 2013. Faecal carriage of extended-spectrum beta-lactamase (ESBL)-producing commensal Klebsiella pneumoniae and Escherichia coli from hospital out-patients in Southern Nigeria. *International Journal of Medicine and Medical Sciences*, *5*(3), pp.97-105.
75. Mulvey, M.R., Bryce, E., Boyd, D., Ofner-Agostini, M., Christianson, S., Simor, A.E. and Paton, S., 2004. Ambler class A extended-spectrum beta-lactamase-producing Escherichia coli and Klebsiella spp. in Canadian hospitals. *Antimicrobial agents and chemotherapy*, *48*(4), pp.1204-1214.
76. Hasani, A., Purmohammad, A., Rezaee, M.A., Hasani, A. and Dadashi, M., 2017. Integron-mediated multidrug and quinolone resistance in extended-spectrum β-lactamase-producing Escherichia coli and Klebsiella pneumoniae. *Archives of Pediatric Infectious Diseases*, *5*(2).
77. Juma, B.W., Kariuki, S., Waiyaki, P.G., Mutugi, M.M. and Bulimo, W.D., 2016. The prevalence of TEM and SHV genes among Extended-Spectrum Beta-Lactamase-producing Klebsiella pneumoniae and Escherichia coli. *African journal of pharmacology and therapeutics*, *5*(1).
78. Hansen, D.S., Schumacher, H., Hansen, F., Stegger, M., Hertz, F.B., Schønning, K., Justesen, U.S., Frimodt-Møller, N. and DANRES Study Group, 2012. Extended-spectrum β-lactamase (ESBL) in Danish clinical isolates of Escherichia coli and Klebsiella pneumoniae: Prevalence, β-lactamase distribution, phylogroups, and co-resistance. *Scandinavian journal of infectious diseases*, *44*(3), pp.174-181.
79. Ushie, S.N., Oyedeji, K.S., Ogban, G.I., Ushie, D.E., Nwaokorie, F.O., Odeniyi, O.M., Ola-Bello, O.I., Okorafor, K.S. and Ezeador, C.O., 2020. Molecular Epidemiology of Extended Spectrum Beta-lactamases Producing Escherichia coli and Klebsiella Species in Catheterized Patients. *European Journal of Medical and Health Sciences*, *2*(4).
80. Yarima, A., Haroun, A.A., Bulus, T. and Manga, M.M., 2020. Occurrence of Extended Spectrum Beta Lactamase Encoding Genes among Urinary Pathogenic Escherichia coli and Klebsiella pneumoniae Isolates Obtained from a Tertiary Hospital in Gombe Nigeria. *Journal of Biosciences and Medicines*, *8*(09), p.42.
81. Adeyankinnu, F.A., Motayo, B.O., Akinduti, A., Akinbo, J., Ogiogwa, J.I., Aboderin, B.W. and Agunlejika, R.A., 2014. A multicenter study of beta-lactamase resistant Escherichia coli and Klebsiella pneumoniae reveals high level chromosome mediated extended spectrum β lactamase resistance in Ogun State, Nigeria. *Interdisciplinary perspectives on infectious diseases*, *2014*.
82. Deji-Agboola, A.M., Olaosebikan, O.R., Adenipekun, E., Osinupebi, O.A. and Olajubu, F.A., 2020. Extended spectrum beta-lactamases (ESBLs)-producing Escherichia coli and Klebsiella pneumoniae among asymptomatic out-patients in a university health centre. *Annals of Health Research*, *6*(2), pp.133-142.
83. Ghenea, A.E., Zlatian, O.M., Cristea, O.M., Ungureanu, A., Mititelu, R.R., Balasoiu, A.T., Vasile, C.M., Salan, A.I., Iliuta, D., Popescu, M. and Udriștoiu, A.L., 2022. TEM, CTX-M, SHV Genes in ESBL-Producing Escherichia coli and Klebsiella pneumoniae Isolated from Clinical Samples in a County Clinical Emergency Hospital Romania-Predominance of CTX-M-15. *Antibiotics*, *11*(4), p.503.
84. Chirindze, L.M., Zimba, T.F., Sekyere, J.O., Govinden, U., Chenia, H.Y., Sundsfjord, A., Essack, S.Y. and Simonsen, G.S., 2018. Faecal colonization of E. coli and Klebsiella spp. producing extended-spectrum beta-lactamases and plasmid-mediated AmpC in Mozambican university students. *BMC infectious diseases*, *18*, pp.1-8.
85. ABE, I.A., KOFFI, M., SOKOURI, P.D., KONAN, T.K., William, Y.A.V.O., TIDOU, S.A. and Nâ, S.P., 2021. Molecular characterization and in silico analysis of mutations associated with extended-spectrum beta-lactamase resistance in uropathogenic Escherichia coli and Klebisiella pneumoniae in two hospitals, Cte dIvoire. *International Journal of Genetics and Molecular Biology*, *13*(1), pp.9-20.
86. Amer, R., El-Baghdady, K., Kamel, I. and El-Shishtawy, H., 2019. Prevalence of extended spectrum Beta-Lactamase Genes among Escherichia coli and Klebsiella pneumoniae clinical isolates. *Egyptian Journal of Microbiology*, *54*(1), pp.91-101.
87. Khanal, L.K., Amatya, R., Sah, A.K., Adhikari, R.P., Khadka, S., Sapkota, J. and Rai, S.K., 2022. Prevalence of Extended Spectrum Beta Lactamase producing Escherichia coli and Klebsiella spp. from urinary specimen in a tertiary care hospital. *Nepal Medical College Journal*, *24*(1), pp.75-80.
88. Karim, M.H., Alam, S.M.D. and Yeasmin, T., 2017. Molecular identification of TEM and SHV genes in extended spectrum beta-lactamase producing Escherichia coli and Klebsiellae pneumoniae isolates in a tertiary care hospital, Bangladesh. *Journal of Pure and Applied Microbiology*, *11*(2), pp.1189-1198.
89. Kim, J., Lim, Y.M., Rheem, I., Lee, Y., Lee, J.C., Seol, S.Y., Lee, Y.C. and Cho, D.T., 2005. CTX-M and SHV-12 β-lactamases are the most common extended-spectrum enzymes in clinical isolates of Escherichia coli and Klebsiella pneumoniae collected from 3 university hospitals within Korea. *FEMS Microbiology letters*, *245*(1), pp.93-98.
90. Lee, J., Pai, H., Kim, Y.K., Kim, N.H., Eun, B.W., Kang, H.J., Park, K.H., Choi, E.H., Shin, H.Y., Kim, E.C. and Lee, H.J., 2007. Control of extended-spectrum β-lactamase-producing Escherichia coli and Klebsiella pneumoniae in a children's hospital by changing antimicrobial agent usage policy. *Journal of antimicrobial Chemotherapy*, *60*(3), pp.629-637.
91. Badri, A.M., Ibrahim, I.T., Mohamed, S.G., Garbi, M.I., Kabbashi, A.S. and Arbab, M.H., 2017. Prevalence of extended spectrum beta lactamase (ESBL) producing Escherichia coli, and Klebsiella pneumoniae isolated from raw milk samples in Al Jazirah state, Sudan. *Mol. Biol*, *7*(1), p.201.
92. Bandyopadhyay, S., Bhattacharyya, D., Samanta, I., Banerjee, J., Habib, M., Dutta, T.K. and Dutt, T., 2021. Characterization of multidrug-resistant biofilm-producing Escherichia coli and Klebsiella pneumoniae in healthy cattle and cattle with diarrhea. *Microbial Drug Resistance*, *27*(11), pp.1457-1469.
93. Chenouf, N.S., Carvalho, I., Messaï, C.R., Ruiz-Ripa, L., Mama, O.M., Titouche, Y., Zitouni, A., Hakem, A. and Torres, C., 2021. Extended Spectrum β-Lactamase-Producing Escherichia coli and Klebsiella pneumoniae from Broiler Liver in the Center of Algeria, with Detection of CTX-M-55 and B2/ST131-CTX-M-15 in Escherichia coli. *Microbial Drug Resistance*, *27*(2), pp.268-276.
94. Chijioke, A., 2022. Nsofor., et al.“Extended-Spectrum Beta-Lactamase-Producing Escherichia coli and Klebsiella pneumoniae among Chickens, Poultry Environment and Retail Meats in Owerri Nigeria”. *EC Microbiology*, *18*, pp.41-51.
95. Eibach, D., Dekker, D., Boahen, K.G., Akenten, C.W., Sarpong, N., Campos, C.B., Berneking, L., Aepfelbacher, M., Krumkamp, R., Owusu-Dabo, E. and May, J., 2018. Extended-spectrum beta-lactamase-producing Escherichia coli and Klebsiella pneumoniae in local and imported poultry meat in Ghana. *Veterinary microbiology*, *217*, pp.7-12.
96. Gundogan, N. and Avci, E., 2013. Prevalence and antibiotic resistance of extended-spectrum beta-lactamase (ESBL) producing Escherichia coli and Klebsiella species isolated from foods of animal origin in Turkey. *African journal of microbiology research*, *7*(31), pp.4059-4064.
97. Jamborova, I., Janecko, N., Halova, D., Sedmik, J., Mezerova, K., Papousek, I., Kutilova, I., Dolejska, M., Cizek, A. and Literak, I., 2018. Molecular characterization of plasmid-mediated AmpC beta-lactamase-and extended-spectrum beta-lactamase-producing Escherichia coli and Klebsiella pneumoniae among corvids (Corvus brachyrhynchos and Corvus corax) roosting in Canada. *FEMS microbiology ecology*, *94*(11), p.fiy166
98. Johansson, V., Nykäsenoja, S., Myllyniemi, A.L., Rossow, H. and Heikinheimo, A., 2022. Genomic characterization of ESBL/AmpC-producing and high-risk clonal lineages of Escherichia coli and Klebsiella pneumoniae in imported dogs with shelter and stray background. *Journal of global antimicrobial resistance*, *30*, pp.183-190.
99. Kuan, N.L., Chang, C.W., Lee, C.A. and Yeh, K.S., 2016. Extended-spectrum beta-lactamase-producing Escherichia coli and Klebsiella pneumoniae isolates from the urine of dogs and cats suspected of urinary tract infection in a veterinary teaching hospital. *Taiwan Veterinary Journal*, *42*(03), pp.143-148.
100. Mbah, M.I. and Anyamene, C.O., Extended spectrum beta lactamase producing Escherichia coli and klebsiella species isolated from layer chicken farms in Jalingo, Nigeria.
101. Montso, K.P., Dlamini, S.B., Kumar, A. and Ateba, C.N., 2019. Antimicrobial resistance factors of extended-spectrum beta-lactamases producing Escherichia coli and Klebsiella pneumoniae isolated from Cattle Farms and Raw Beef in North-West Province, South Africa. *BioMed research international*, *2019*.
102. Mwanginde, L.W., Majigo, M. and Kajeguka, D.C., 2021. High carriage rate of extended-spectrum β-lactamase-producing Escherichia coli and Klebsiella species among poultry meat vendors in Dar es Salaam: the urgent need for intervention to prevent the spread of multidrug-resistant pathogens. *International Journal of Microbiology*, *2021*.
103. Sai’du, A.S., Apollos, R.P., Mohammed, S., Ejeh, F.E., Tijjani, A.O., Ahmed, B. and Wafar, E., 2022. Microbial Quality and Phenotypic Profile of Extended Spectrum Beta–‎ Lactamase Producing Escherichia coli and Klebsiella species Contamination‎ in Dressed Chicken Meat in Maiduguri Metropolis, Northeastern Nigeria. *Sahel Journal of Veterinary Sciences*, *19*(1), pp.22-30.
104. Saidani, M., Messadi, L., Mefteh, J., Chaouechi, A., Soudani, A., Selmi, R., Dâaloul-Jedidi, M., Chehida, F.B., Mamlouk, A., Jemli, M.H. and Madec, J.Y., 2019. Various Inc-type plasmids and lineages of Escherichia coli and Klebsiella pneumoniae spreading blaCTX-M-15, blaCTX-M-1 and mcr-1 genes in camels in Tunisia. *Journal of Global Antimicrobial Resistance*, *19*, pp.280-283.
105. Sivaraman, G.K., Rajan, V., Vijayan, A., Elangovan, R., Prendiville, A. and Bachmann, T.T., 2021. Antibiotic resistance profiles and molecular characteristics of extended-spectrum beta-lactamase (ESBL)-producing Escherichia coli and Klebsiella pneumoniae isolated from shrimp aquaculture farms in Kerala, India. *Frontiers in Microbiology*, *12*, p.622891.
106. Vo, A.T., van Duijkeren, E., Fluit, A.C. and Gaastra, W., 2007. Characteristics of extended-spectrum cephalosporin-resistant Escherichia coli and Klebsiella pneumoniae isolates from horses. *Veterinary microbiology*, *124*(3-4), pp.248-255.
107. Atta, H.I., Idris, S.M., Gulumbe, B.H. and Awoniyi, O.J., 2022. Detection of extended spectrum beta-lactamase genes in strains of Escherichia coli and Klebsiella pneumoniae isolated from recreational water and tertiary hospital waste water in Zaria, Nigeria. *International Journal of Environmental Health Research*, *32*(9), pp.2074-2082.
108. Devi, L.S., Broor, S., Chakravarti, A. and Chattopadhya, D., 2020. Livestock manure as potential reservoir of CTX-M type extended-spectrum β-lactamase producing Escherichia coli and Klebsiella pneumoniae associated with carbapenemase production. *J Pure Appl Microbiol*, *14*(1), pp.171-181.
109. Hassen, B., Abbassi, M.S., Benlabidi, S., Ruiz-Ripa, L., Mama, O.M., Ibrahim, C., Hassen, A., Hammami, S. and Torres, C., 2020. Genetic characterization of ESBL-producing Escherichia coli and Klebsiella pneumoniae isolated from wastewater and river water in Tunisia: predominance of CTX-M-15 and high genetic diversity. *Environmental Science and Pollution Research*, *27*, pp.44368-44377.
110. Mahato, S., Mahato, A., Pokharel, E. and Tamrakar, A., 2019. Detection of extended-spectrum beta-lactamase-producing E. coli and Klebsiella spp. in effluents of different hospitals sewage in Biratnagar, Nepal. *BMC research notes*, *12*(1), pp.1-6.
111. Romyasamit, C., Sornsenee, P., Chimplee, S., Yuwalaksanakun, S., Wongprot, D. and Saengsuwan, P., 2021. Prevalence and characterization of extended-spectrum β-lactamase-producing Escherichia coli and Klebsiella pneumoniae isolated from raw vegetables retailed in Southern Thailand. *PeerJ*, *9*, p.e11787.
